# Circular RNA expression is abundant and correlated to aggressiveness in early-stage bladder cancer

**DOI:** 10.1038/s41525-017-0038-z

**Published:** 2017-11-28

**Authors:** Trine Line Hauge Okholm, Morten Muhlig Nielsen, Mark P. Hamilton, Lise-Lotte Christensen, Søren Vang, Jakob Hedegaard, Thomas Birkballe Hansen, Jørgen Kjems, Lars Dyrskjøt, Jakob Skou Pedersen

**Affiliations:** 1 Department of Molecular Medicine (MOMA), Aarhus University Hospital, Aarhus, 8200 Denmark; 2 Department of Molecular and Cellular Biology, Baylor College of Medicine, Houston, TX 77030 USA; 3 Department of Molecular Biology and Genetics (MBG), and Interdisciplinary Nanoscience Center (iNANO), Aarhus University, Aarhus, 8000 Denmark; 4 Bioinformatics Research Center (BiRC), Aarhus University, Aarhus, 8000 Denmark

## Abstract

The functions and biomarker potential of circular RNAs (circRNAs) in various cancer types are a rising field of study, as emerging evidence relates circRNAs to tumorigenesis. Here, we profiled the expression of circRNAs in 457 tumors from patients with non-muscle-invasive bladder cancer (NMIBC). We show that a set of highly expressed circRNAs have conserved core splice sites, are associated with Alu repeats, and enriched with Synonymous Constraint Elements as well as microRNA target sites. We identified 113 abundant circRNAs that are differentially expressed between high and low-risk tumor subtypes. Analysis of progression-free survival revealed 13 circRNAs, among them circHIPK3 and circCDYL, where expression correlated with progression independently of the linear transcript and the host gene. In summary, our results demonstrate that abundant circRNAs possess multiple biological features, distinguishing them from low-expressed circRNAs and non-circularized exons, and suggest that circRNAs might serve as a new class of prognostic biomarkers in NMIBC.

## Bladder cancer: Circular RNAs show prognostic biomarker potential

Expression levels of non-coding “circular” RNA molecules could be used as a prognostic biomarker for patients with early-stage bladder cancer. A team led by Trine Line Hauge Okholm and Jakob Skou Pedersen from Aarhus University Hospital, Denmark, profiled the expression of these loop-forming, potentially gene-regulating RNAs in biopsied tumor samples from 457 patients with bladder cancer that had not invaded nearby muscle tissue. They identified a suite of 113 circular RNAs that were abundant and differentially expressed between patients with different molecular subtypes of bladder cancer. The researchers also found a smaller set of 13 circular RNAs for which expression levels correlated with disease progression. These non-coding RNA molecules, by indicating likely patient outcomes, could potentially serve as future diagnostic aids to inform treatment strategies and decisions.

## Introduction

Circular RNA (circRNA) derived from precursor mRNA is a large class of non-coding RNA that was first identified in the early 1990s.^1^ Since then, thousands of circRNAs in mammalian cells have been reported, some of which are highly abundant and conserved among species.^2–4^ They are characterized by a covalently closed circular structure, formed in a process where a downstream donor splice site “backsplices” to an upstream acceptor splice site.^5^ Two distinct paths have been proposed for circRNA formation: an exon-skipping event where the skipped exons undergo internal splicing more rapidly than debranching and an intron-pairing-driven circularization, where circRNAs are formed by intronic motifs, e.g., Alu repeats, that pair up and position splice sites in close proximity.^6^

The functions of circRNAs is still largely unexplored. Some well-studied circRNAs are able to sponge microRNAs (miRNAs) as shown for ciRS-7, circ-SRY, and circHIPK3.^2,7,8^ Other circRNAs can interact with RNA-binding proteins, such as circMbl that affects splicing by binding Mbl protein^9^ and circFoxo3 that blocks cell cycle progression by forming a ternary complex with p21 and CDK2.^10^ Recently, studies have suggested that some circRNAs are translated into proteins, e.g., circZNF609 and circMbl3.^11,12^ Other examples have been reviewed recently.^13,14^

Early studies showed that circRNAs are preferentially located in the cytoplasm^6,15^ and due to their circular structure, circRNAs are very stable as they are not degraded by RNA exonucleases.^6^ A recent study has shown that cells can excrete circRNAs into extracellular vesicles, where circRNAs, e.g., circHIPK3, are enriched over their linear counterparts.^16^ Serum from tumor-bearing mice have revealed abundant circRNAs in exosomes that are differentially expressed between cancer patients and healthy controls.^17^ In addition, hundreds of circRNAs have been reported at higher levels in human blood than corresponding linear mRNAs.^18^

Bladder cancer (BC) is the ninth most common cancer type in the world with 430,000 new cases and 165,000 deaths in 2012.^19^ Survival rate depends on the stage of the cancer at diagnosis. Patients diagnosed with non-muscle-invasive bladder cancer (NMIBC) have a 5-year survival rate of ~90%.^20^ In contrast, patients with muscle-invasive BC (MIBC) have a survival rate of ~50%,^21^ which further drops to ~5% in the presence of distant metastasis.^22^ Since patients diagnosed with NMIBC are routinely monitored due to the risk of tumor recurrence and progression, BC is one of the most expensive cancer types to treat measured on a per-patient cost from diagnosis to death.^23^ Identification of biomarkers that can predict the outcome of patients diagnosed with NMIBC, e.g., disease recurrence, progression, and death, would thus be cost effective and beneficial to clinicians in order to improve prognosis and treatment response of patients.

**Fig. 1:**
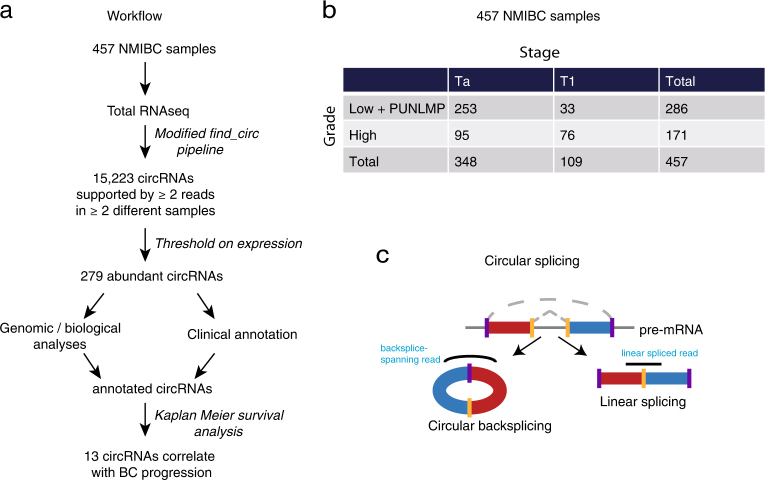
Workflow and data set. **a** Circular RNA expression was analyzed in total RNA from 457 NMIBC samples using a modified version of the *find_circ* pipeline (see Methods). In total, 38,365 unique circRNAs were identified and characterized. A total of 279 highly expressed circRNAs were analyzed and annotated with their biological features and clinical correlations. A set of circRNA candidates that correlate with bladder cancer progression were identified. **b** Distribution among stages and grades of the 457 bladder cancer samples. **c** Illustration of how alternative splicing can generate circular transcripts (left) instead of linear transcripts (right). The positions of reads revealing a backspliced circRNA versus a linearly spliced transcript are shown

Due to their structural stability, specificity, and accessibility, circRNAs may represent an attractive new class of biomarkers. Consequently, the role of circRNAs in tumor development and their biomarker potential have been studied across various cancer types.^24–28^ However, the overall expression profile of circRNAs in early-stage BC has not been thoroughly addressed and remains unclear. Here we characterize the expression of circRNAs in a large cohort of 457 NMIBC samples and demonstrate that a subset of highly expressed circRNAs are enriched with a number of biological features, distinguishing them from linearly spliced exons (Fig. 1a). Additionally, we show that a subset of these correlate with BC progression independently of the corresponding linear transcript and the parent gene. We conclude that a subset of circRNAs, among these circHIPK3 and circCDYL, are potentially prognostic biomarkers for patients diagnosed with early-stage BC.

## Results

### Profiling of circRNAs in human BC samples

**Fig. 2:**
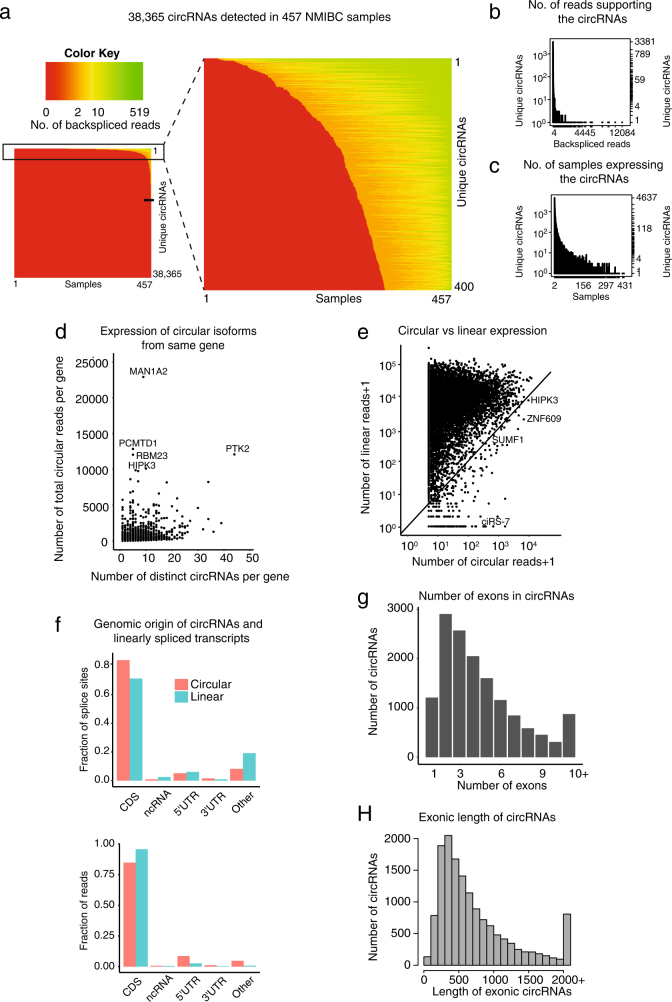
Profiling of circular RNAs in human bladder cancer. **a** Heatmap of all detected circRNAs (supported by ≥two reads, *n* = 38,365) and their expression level across samples. Rows represent unique circRNAs and have been sorted by number of samples with expression of each. Samples are sorted by circRNA expression level within each row. Black tick mark indicates 20,819 unique circRNAs that are only supported by two reads in one sample. The small heatmap (right) gives and overview of all samples and the larger heatmap (right) shows the expression distributions for the 400 most abundantly expressed circRNAs. **b** The number of backspliced reads supporting distinct circRNAs (supported by ≥ two reads in ≥ two different samples, *n* = 15,223). *Y*-axis is plotted on a logarithmic scale (log10). **c** The number of samples expressing distinct circRNAs. *Y*-axis is plotted on a logarithmic scale (log10). **d** Number of total circular reads per gene versus number of distinct circRNAs per gene. **e** Number of circular and corresponding linear reads supporting each candidate. *X*-axis and *Y*-axis are plotted on a logarithmic scale (log10). There are 303 circRNAs with no support of linear splicing, including ciRS-7. **f** Genomic origin of bladder cancer circRNAs and linearly spliced transcripts. Upper panel shows fraction of splice sites and lower panel shows fraction of reads and hence combined expression level of circRNAs from each region. Relative levels of circular (red) and linear (blue) splicing can be compared. 3′-UTR: 3′-untranslated region, 5′-UTR: 5′-untranslated region, CDS: coding sequence, ncRNA: non-coding RNA. **g** Number of exons composing exonic circRNAs (*n* = 14,569). **h** Exonic length of circRNAs (*n* = 14,569)

We characterized circRNA transcripts using whole transcriptome RNA-Seq data from 457 NMIBC samples (348 Ta and 109 T1, Fig. 1b, Supplementary Table 1).^29^ A modified version of the *find_circ* pipeline^2,30^ with an increased filtering stringency^2,30^ (see Methods) was used to identify backsplice-spanning reads (Fig. 1c). In total, 38,365 unique circRNAs supported by at least two reads were identified, of which hundreds are expressed at high levels across many samples (Fig. 2a). However, many of these may be false positives, as 20,819 unique circRNAs are only supported by two reads (above black tick mark on Fig. 2a) and 23,142 circRNAs are only expressed in one sample. We used a more robust criteria to identify 15,223 unique circRNAs that are supported by at least two reads in at least two different samples (Fig. 2b). We used this set for all downstream analyses.

We analyzed the expression of circRNAs per gene and found profound variation in the expression profiles; some genes give rise to many circRNAs, while other genes give rise to few circRNAs at high frequency, e.g., *MAN1A2* (Fig. 2d). We also found that some circular transcripts are more expressed than their linear counterpart (503/15,223) (Fig. 2e).

We annotated the genomic origin of circularly and linearly spliced transcripts (see Methods) with respect to their splice site and read coverage. We observed that most splice sites of circRNAs overlap protein-coding exons (84.1%), whereas smaller fractions of circRNAs are derived from non-coding RNAs (0.9%), 5′-untranslated regions (UTRs) (5.1%), and 3′-UTRs (1.6%) (Fig. 2f, upper panel). The origin of splice sites of linearly spliced transcripts (protein-coding exons: 71.4%, non-coding RNAs: 2.5%, 5′-UTRs: 6%, and 3′-UTRs: 0.9%) were significantly different from the origin of splice sites of circRNAs (*P* < 2.2e-16, *χ* ^2^ test, Fig. 2f, upper panel). In addition, the read fraction that are annotated to each genomic location are significantly different between the circularly and linearly spliced transcripts (*P* < 2.2e-16, *χ* ^2^ test, Fig. 2f, lower panel). Most exonic circRNAs contain less than five exons (Fig. 2g) and have a median exonic length of 553 bp (Fig. 2h, *n* = 14,569).

In order to validate that the predicted circRNAs are actually circular transcripts, we designed divergent primers spanning the backsplice junctions of five different circRNAs expressed at various levels. Sanger sequencing of the polymerase chain reaction (PCR) product confirmed that the transcripts are indeed circular (Supplementary Fig. 1a–e).

### Characterizing circRNAs in BC

**Fig. 3:**
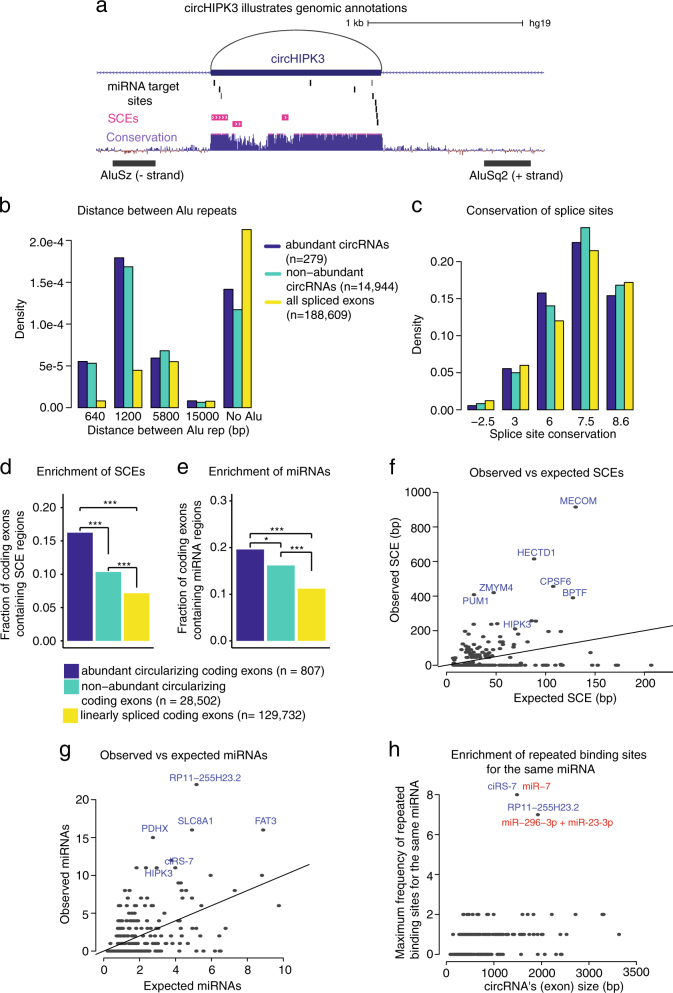
Abundant circRNAs are highly conserved and enriched with Alu repeats, synonymous constraint elements (SCEs), and putative miRNA binding sites. **a** Genomic location of circHIPK3 illustrates genomic annotations, incl. miRNA target sites (black bars), SCEs (purple), evolutionary conservation level (blue), and Alu repeat elements in inverted directions (black). **b** Density plots of the distance between the closest pair of inverted homologous Alu repeats surrounding abundant circRNAs, non-abundant circRNAs, and linearly spliced exons, omitting the size of the circRNAs or spliced exons themselves. **c** Density plots of the average PhyloP position-specific conservation score of the four core nucleotides involved in splicing. **d**,** e** The fraction of linearly spliced exons, non-abundant circularized coding exons, and abundant circularized coding exons that contain **d** SCEs and **e** conserved miRNA binding regions. *P < 0.5, ***P < 0.001 (*χ* ^2^ test). **f** Expected versus observed SCE length of overlap for abundant circRNAs composed of coding exons (*n* = 243). **g** Expected versus observed number of miRNA target sites for abundant circRNAs (exonic circRNAs and all non-exonic circRNAs shorter than 10,000 bp were included; *n* = 270). **h** The maximal number of binding sites for individual miRNAs within each circRNA (exonic circRNAs and all non-exonic circRNAs shorter than 10,000 bp were included, *n* = 270). The miRNA with most binding sites are noted for the two top-ranked circRNAs

To characterize the genomic and biological features of circRNAs in BC, we annotated circRNAs and all spliced exons with their distance to Alu repeats, conservation of core splice sites, enrichment of both synonymous constraint elements (SCEs), and putative miRNA binding sites. To identify circRNAs that are highly expressed in a large fraction of BC samples, we defined a set of circRNAs that are expressed in at least 30 samples and supported by at least 20 reads in at least one sample. We contrasted the statistical properties from the genomic analyses of these abundant circRNAs (*n* = 279, Supplementary Table 2) with non-abundant circRNAs (*n* = 14,944) and with all spliced exons in the genome (*n* = 188,609). The circHIPK3 locus exemplifies the use of the annotations (Fig. 3a).

At first, we investigated whether circRNAs are enriched with inverted homologous Alu repeats, which are involved in the biogenesis of many circRNAs.^6^ We looked for the nearest pair of inverted homologous Alu repeats within a window of 20 kb surrounding circRNAs as well as all spliced exons (see Methods). In agreement with previous reports,^6^ we found that both abundant and non-abundant circRNAs are more likely to be in close proximity to inverted homologous Alu repeats than exons in general, and less likely to have no surrounding Alu repeats in the interrogated window (*P* < 0.001, Wilcoxon rank sum test, Fig. 3b, Supplementary Fig. 2a).

Next, we evaluated and contrasted the evolutionary conservation of backspliced circRNA splice sites with linearly spliced exons. High levels of conservation reflect strong functional constraints through evolution, which could stem from maintenance of backsplicing and functional circRNAs. We evaluated the conservation of splice sites by averaging the site-specific PhyloP conservation scores of the four nucleotides involved in the two core splice sites. The results showed that splice sites of abundant circRNAs (mean PhyloP score = 6.15) and non-abundant circRNAs (mean PhyloP score = 6.15) are more conserved than the splice sites of all spliced exons (mean PhyloP score = 5.8) (*P* < 0.001 between non-abundant circRNAs and all spliced exons, Wilcoxon rank sum test, Fig. 3c, Supplementary Fig. 2b).

CircRNAs have been proposed to act as miRNA sponges.^2,7^ For this reason, we explored the presence of SCEs, which are protein-coding sequences that are under selection for additional, overlapping functions, e.g., miRNA target sites or binding sites for proteins.^31^ The SCE analyses were performed for individual protein-coding exons (see Methods). We found that the fraction of coding exons that contain SCEs is significantly enriched for abundant circularized coding exons (*P* < 0.001 for all pairwise comparisons, *χ* ^2^ test, Fig. 3d). In addition, abundant circular coding exons have a higher SCE coverage than non-abundant circular coding exons and linearly spliced coding exons (*P* < 0.01 for all pairwise comparisons, Kolmogorov–Smirnov test, Supplementary Fig. 2c). This was not due to a difference in exon size (not significant, Wilcoxon rank sum test, Supplementary Fig. 2d) as abundant circularized coding exons also show a higher SCE coverage per base pair (bp) (*P* < 0.001, Wilcoxon rank sum test, Supplementary Fig. 2e).

Finally, we examined the enrichment of conserved miRNA binding sites by analyzing Ago associated exons based on CLIP data^32^ (see Methods). The fraction of abundant circularized coding exons that contain miRNA binding sites is significantly higher than the fraction of non-abundant circularized coding exons and linearly spliced coding exons (*P* < 0.05 for all pairwise comparisons, *χ* ^2^ test, Fig. 3e). Additionally, highly expressed circularized coding exons have a higher coverage of miRNA targets than linearly spliced exons (*P* < 0.001, Kolmogorov–Smirnov Test, Supplementary Fig. 2f) and a higher coverage of miRNA targets per bp (*P* < 0.05 for all pairwise comparisons, Wilcoxon rank sum test, Supplementary Fig. 2g).

To identify individual abundant circRNAs with a high enrichment of SCEs and miRNA binding sites, we calculated the expected coverage of SCEs and expected number of miRNA binding sites, controlling for exon size (see Methods). We observed 72 circRNAs that contain more SCEs than expected, among these circHIPK3 (Fig. 3f, *n* = 243). Additionally, we found 92 circRNAs that are enriched with more putative miRNA target sites than expected, e.g., circHIPK3, ciRS-7, and circRNAs arising from the genes *RP11-255H23.2* and *SLC8A1* (Fig. 3g, circRNAs longer than 10 kb were not considered here, *n* = 270). Further, to explore if the abundant circRNAs could act as sponges, we evaluated whether they were enriched with repeated binding sites for the same miRNA. As expected, ciRS-7 shows the highest binding frequency of the same miRNA with eight miR-7 target sites in brain tissue (Fig. 3h, Supplementary Table 3). In addition, we identify a circRNA overlapping the pseudogene *RP11-255H23.2*, that contain seven potential miRNA target sites for miR-23-3p and miR-296-3p, respectively, in huPancreas and HeLa cells (Fig. 3h, Supplementary Table 3). We identify no other circRNAs that are enriched with more than two binding sites for the same miRNA, indicating that even though abundant circRNAs are enriched with miRNA target sites, miRNA-sponging of individual miRNAs is not the general function of the circRNAs studied here.

Studies have shown that the biogenesis of some circRNAs are regulated by ADAR1 and Quaking. ADAR1 antagonizes circularization by editing A-residues to inosines and thereby melting the stem structure that is generated by reverse complementary matches like Alu-repeats.^33^ Quaking, on the other hand, promotes circRNA formation during human epithelial–mesenchymal transition by binding to circRNA exons flanked by intronic Quaking binding motifs.^34^ We find that *Quaking* expression correlate positively with the overall expression of circRNAs across samples (Spearman’s rho = 0.323, *P* = 1.86e-12, Supplementary Fig. 3a) whereas *ADAR1* expression does not (Spearman’s rho = 0.0568, *P* = 0.226, Supplementary Fig. 3b). Among the abundant circRNAs, we find 32 circRNAs that correlate negatively or positively with *ADAR1* expression and 231 circRNAs that correlate positively with *Quaking* expression (false discovery rate (FDR) < 0.1, Spearman correlation with Benjamini–Hochberg (BH) correction, Supplementary Fig. 3c, d). We found no association between circRNA-*ADAR1* correlation and the distance between Alu repeats (Spearman’s rho = −0.026, *P* = 0.667, data not shown).

### Abundant circRNAs show differential expression between NMIBC classes

**Fig. 4:**
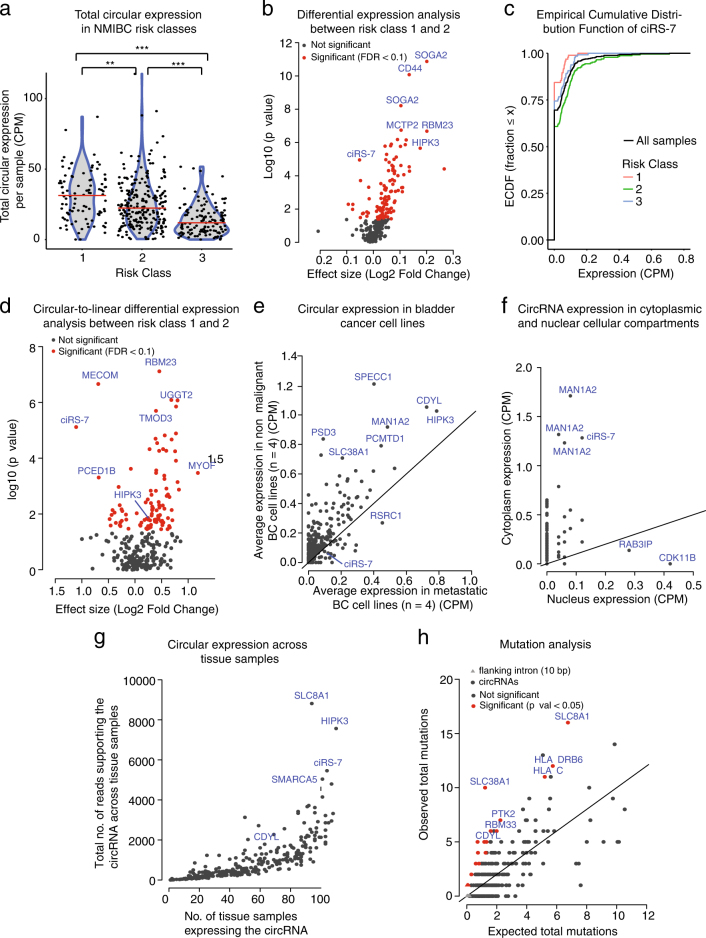
Multiple abundant circRNAs are differentially expressed between NMIBC risk classes. **a** Total sample-wise circRNA expression. Red line indicates median expression. **P < 0.01; ***P < 0.001 (Wilcoxon rank sum test). **b** Differential expression analysis of 279 abundant circRNAs between risk class 1 and 2 (Wilcoxon rank sum test). The log2 Fold Changes are plotted against the negative log10 *P*-values. Colors indicate if observed changes are significant (red) or not (grey) (BH correction with FDR < 0.1). **c** Plot of the empirical cumulative distribution function of ciRS-7 illustrates bladder cancer heterogeneity, where only a subset of samples show any expression. **d** Differential expression analysis of circular-to-linear ratios of abundant circRNAs (*n* = 279) between risk class 1 and 2 (Wilcoxon rank sum test). Circular-to-linear ratios are obtained by dividing the number of circular reads with the number of linear reads. The log2 Fold Changes are plotted against the negative log10 *P*-values. Colors indicate if observed changes are significant (red) or not (grey) (BH correction with FDR < 0.1). **e** Average circular expression of abundant circRNAs (*n* = 252) in four non-malignant bladder cancer (BC) cell lines (Nhutert, HCV29, HT1197, and RT4) and four metastatic BC cell lines (J82, HT-1376, T24, and UMUC3). **f** Circular expression of abundant circRNAs (*n* = 160) in the the cytoplasmic and nuclear cellular compartments of the cell line FL3. **g** Expression of abundant circRNAs (*n* = 276) across 113 tissue samples. Number of tissue samples expressing the circRNA versus total number of reads supporting the circRNA across all tissue samples. **h** Mutation analysis of abundant circRNAs and intronic flanking regions by ncdDetect across a cohort of 507 pan-cancer genomes. CircRNAs on chromosome X and Y were not evaluated. Observed number of mutations versus expected number of mutations. The entire circRNA region as well as the first 10 bp of the intronic flanking regions were analyzed. Shapes indicate flanking intronic regions (triangle) and circRNAs (circles). Colors indicate if observed changes are nominally significant (red) or not (grey) (*P* < 0.05, ncdDetect)

Next, we investigated the clinical correlations of the 279 abundant circRNAs’ expression levels. In a previous study of this data set, three major subclasses of NMIBC with molecularly distinct characteristics and different clinical outcome were identified.^29^ It was found that class 1 (*n* = 96) and class 2 (*n* = 232) showed luminal-like characteristics while class 3 (*n* = 129) showed basal-like characteristics. We observed, that the total circular expression per sample is significantly different between the three classes, decreasing from risk class 1 to 3 (*P* < 0.001 for all pairwise comparisons, Wilcoxon rank sum test, Fig. 4a). Overall, class 1 tumors are characterized by a good prognosis while class 2 tumors are characterized by a poor prognosis.^29^ The underlying biological characteristics of class 3 tumors are poorly defined, we thus focused on class 1 versus class 2. We identified 113 circRNAs that were differentially expressed between class 1 and 2, among these a circRNA from *CD44*, circHIPK3, and ciRS-7 (FDR < 0.1, Wilcoxon rank sum test with BH correction). Most differentially expressed circRNAs (88%) are upregulated in class 1 compared to the tumors predicted to be more aggressive, class 2 (Fig. 4b). Yet, we identified a few circRNAs that were upregulated in class 2. Of particular interest, one of these is ciRS-7, which has been shown to sponge the tumor suppressor microRNA, miR-7, and thereby prevent repression of its oncogenic targets.^7,35^

BC is a molecularly heterogeneous disease,^36^ and we find that the circRNA expression is also highly variable within prognostic subclasses. Generally, when we look at the expression of individual circRNAs, we observe many samples with zero reads and a subset with high circRNA expression (Fig. 4c).

Since the differentially expressed circRNAs could be a reflection of the linear counterparts, we performed differential expression analysis on the circular-to-linear (circ-to-lin) ratios. We identified 102 circRNAs that show perturbed expression profiles between class 1 and 2 independently of the linear transcript (FDR < 0.1, Wilcoxon rank sum test with BH correction, Fig. 4d).

### Expression of abundant circRNAs in BC cell lines and across 113 tissue samples

We analyzed the expression of all of the detectable abundant circRNAs (*n* = 252) in whole transcriptome RNA-Seq data from eight BC cell lines (Nhutert, HCV29, HT1197, RT4, J82, HT-1376, T24, and UMUC3).^29^ Consistent with the expression profiles between risk classes, most circRNAs (216/252) were expressed at higher levels in the non-malignant BC cell lines compared to the metastatic BC cell lines, e.g., a circRNA overlapping the *SPECC1* gene, circCDYL, and circHIPK3 (Fig. 4e). Accordingly, ciRS-7 was found at higher levels in metastatic BC cell lines. No circRNAs were differentially expressed between non-malignant and metastatic BC cell lines after multiple testing correction (FDR < 0.1, Wilcoxon rank sum test).

We performed RNA-Seq of total RNA and of poly(A) selected RNA from three fractionated cell lines; two BC cell lines (T24 and HCV29) and a T24 derivate (FL3) from a lung metastasis (GSE100971) (see Methods). We analyzed the expression of the abundant circRNAs, and found that almost all of the detectable abundant circRNAs were more expressed in cytoplasm than in nucleus (FL3: 157/160, T24: 132/133, and HCV29: 92/93) (Fig. 4f, Supplementary Fig. 4). In all three cell lines, circular isoforms from *MAN1A2* and ciRS-7 were some of the highest expressed circles in the cytoplasm.

Further, we evaluated the abundance and specificity of our candidates across other tissues in 113 human adult and fetal total RNA-Seq samples from ENCODE,^37^ some of which have previously been used to identify tissue-specific circRNAs.^38^ By using the *find_circ* pipeline, we profiled the expression of detectable abundant circRNAs (*n* = 276) across all tissue samples (Supplementary Fig. 5). A circRNA overlapping *SLC8A1*, circHIPK3, and ciRS-7 are the circRNAs that are highest expressed across all tissue samples (Fig. 4g).

### Mutation analysis of abundant circRNAs

We next asked if the circRNA expression perturbations between BC risk classes appeared to be caused by mutations. In general, cancer develops by the accumulation of mutations that disrupt key cellular functions. Most mutations are harmless, so called passenger-mutations, but some mutations are driver-mutations that affect a gene or regulatory element. These perturb normal cellular function and provide growth advantages for cancer cells. We addressed the presence of driver-mutations in circRNAs using ncdDetect,^39^ which compares the observed number of mutations to a neutral sample-specific and position-specific background model. The analysis was performed on a pan-cancer set of 505 whole genome cancer samples.^40^ We analyzed the entire circRNA transcript as well as the first 10 bp of the intronic flanking regions to evaluate mutations at or near the splice sites. We do not see many mutations in the short intronic flanking regions, but we observed some circRNAs that contain more mutations than expected, e.g., circRNAs arising from *SLC8A1, SLC38A1*, and circCDYL (Fig. 4h). However, after BH correction none were significant, suggesting that the observed circRNA expression perturbation is not caused by high-frequency pan-cancer driver mutations.

### Expression of 13 circRNAs correlated with BC progression

**Fig. 5:**
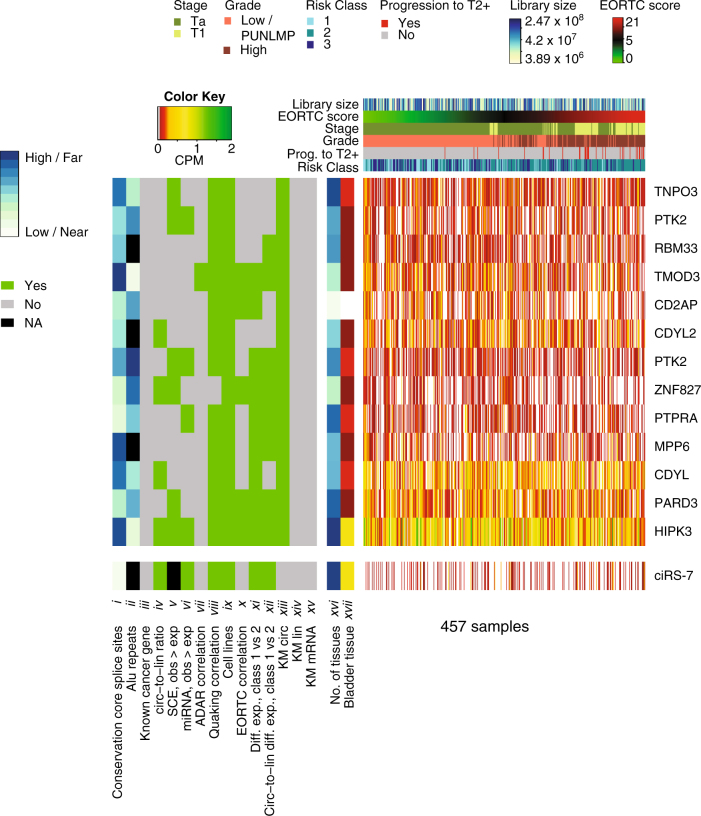
13 circRNAs correlate with bladder cancer progression. Heatmap of 13 circRNAs that correlate with bladder cancer progression independently of the linear transcript and the mRNA, together with ciRS-7. Expression levels in counts per million (CPM) are denoted with a color gradient (right part). White denotes no reads. Samples are ordered according to their EORTC score. For each sample, colored bars above expression heatmap denote stage, grade, prognostic class, library size, and EORTC score and whether or not it progresses to muscle-invasive bladder cancer (T2+). Properties of individual circRNAs are denoted with larger colored bars (left of expression heatmap). Properties that can be represented by Yes/No/NA: (*iii*) Known cancer gene (does the circRNA arise from a known cancer gene); (*iv*) Circ-to-lin ratio > 1 (is the average circular-to-linear expression level across all samples greater than 1); (*v*) SCE, obs > exp (is the observed SCE coverage higher than expected SCE coverage based on exon size); (*vi*) miRNA, obs > exp (is the observed miRNA coverage higher than expected miRNA coverage based on exon size); (*vii*) ADAR correlation (is circRNA expression correlated with ADAR expression, Spearman correlation); (*viii*) Quaking correlation (is circRNA expression correlated with Quaking expression, Spearman correlation); (*ix*) Cell lines (are the circRNAs detected in at least one bladder cancer cell line); (*x*) EORTC correlation (is circRNA expression correlated with EORTC score, Spearman correlation); (*xi*) Diff. exp., class 1 versus 2 (differential expression analysis between class 1 and 2, Wilcoxon rank sum test); (*xii*) circ-to-lin diff. exp., class 1 versus 2 (differential expression analysis of the circular-to-linear ratio between class 1 and 2); (*xiii*) KM circ (Kaplan–Meier analysis of the circular transcript), (*xiv*) KM lin (Kaplan–Meier analysis of the corresponding linear transcript); (*xv*) KM mRNA (Kaplan–Meier analysis of the parent gene (mRNA)). The Benjamini–Hochberg procedure were used for multiple testing correction and FDR < 0.1 were declared significant. Properties that range high/far to low/near: (*i*) Conservation core splice sites (average conservation of the four core nucleotides involved in splicing); (*ii*) Alu repeats (the distance between the pair of inverted homologous Alu repeats in closest proximity with the circle); (*xvi*) No. of tissues (in how many of the 113 tissues is the circRNA expressed). (*xvii*) Expression levels in CPM in bladder tissue are denoted with the same color gradient as the heatmap

To identify circRNAs with prognostic potential, patients were divided into two groups based on median expression. We then performed Kaplan–Meier survival analyses on the circular expression, the expression of the corresponding linear transcript that uses the same splice sites as the circRNA, and the corresponding overall mRNA expression. Interestingly, we identified 13 circRNAs that correlate with BC progression independently of the corresponding linear transcript and the parent gene (FDR < 0.1, log-rank test with BH correction, Fig. 5).

The European Organization for Research and Treatment of Cancer (EORTC) score is a generally used scoring system for predicting recurrence and progression of NMIBC.^41^ High stage, high grade, and class 2 is positively associated with a high EORTC score. We used this score to sort the samples in the heatmap of the thirteen circRNAs that correlate independently with progression (Fig. 5) and of all 279 abundant circRNAs (Supplementary Fig. 6). The circRNAs are generally downregulated in class 2 compared to class 1 and accordingly negatively correlated with the EORTC score (FDR < 0.1, Spearman’s Correlation with BH correction, Supplementary Fig. 7).

Of the 13 circRNAs that correlated with cancer progression; nine were differentially expressed between class 1 and 2; four were more expressed in the circular than linear form; and none were derived from known cancer genes^42^ (Supplementary Table 4). Twelve of these circRNAs correlated with *Quaking* expression, seven had more conserved core splice sites (upper quartiles), and ten contained inverted homologous Alu repeats in close proximity to the circle, e.g., circHIPK3 and circCDYL (Supplementary Table 4).

**Fig. 6:**
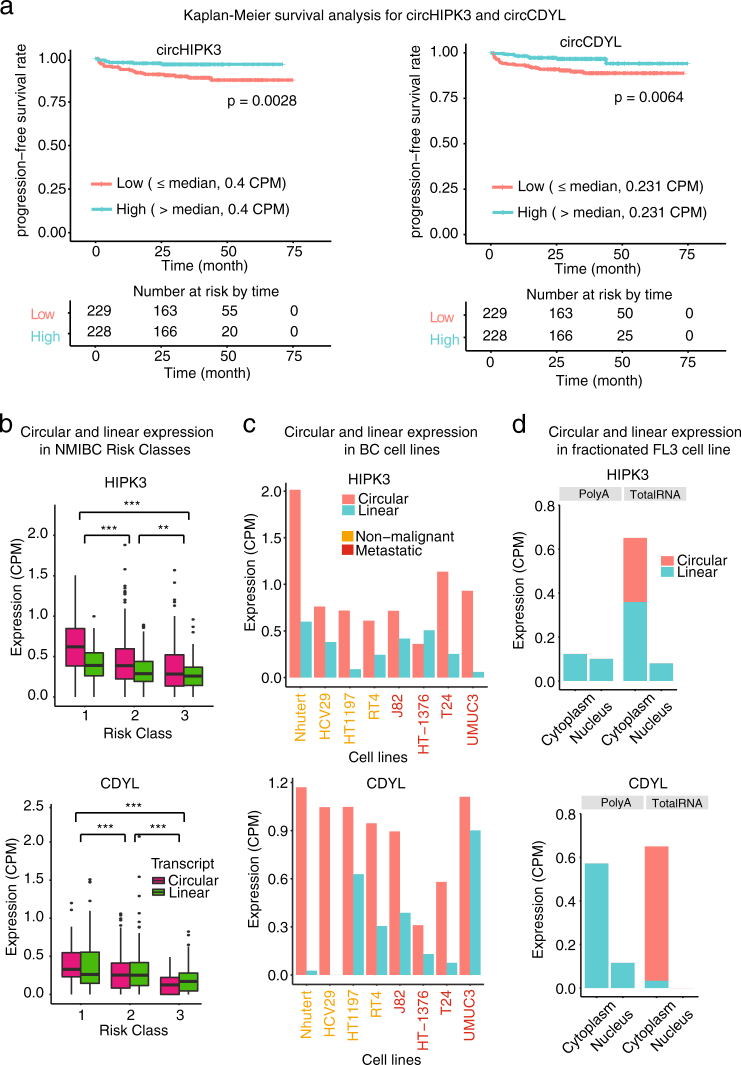
CircHIPK3 and circCDYL are associated with bladder cancer progression. **a** Kaplan–Meier progression plots of circHIPK3 and circCDYL. Median expression used as cutoff. *P*-values obtained by log-rank test. **b** Circular and linear expression of HIPK3 and CDYL in 457 NMIBC samples. Differential expression performed on circular-to-linear ratios; **P < 0.01, ***P < 0.001 (Wilcoxon rank sum test). **c** Circular and linear expression of HIPK3 and CDYL in four non-malignant BC cell lines (Nhutert, HCV29, HT1197, and RT4) and four metastatic BC cell lines (J82, HT-1376, T24, and UMUC3). **d** Circular and linear expression of HIPK3 and CDYL in the cytoplasm and nucleus of FL3. Expression levels are measures based on either poly(A) selected RNA-Seq (poly(A); only mRNAs) or total RNA (both mRNAs and circRNAs)

Both circHIPK3 and circCDYL are promising candidates that possess most of the studied biological and clinical signals. CircHIPK3 is formed from the second exon of *HIPK3*. Our genomic analyses showed that it contains an enrichment of miRNA target sites and SCEs (Fig. 3a). We did not see CLIP data evidence that circHIPK3 is enriched with binding sites for the same miRNA and thereby acts as a miRNA sponge (Fig. 3h). CircCDYL is a less studied circRNA that resembles circHIPK3 in various ways. It consists of one exon (exon four from *CDYL*), the splice sites are highly conserved and inverted homologous Alu repeats are situated nearby. However, neither SCEs nor conserved miRNA binding sites were found in circCDYL. Notably, Kaplan–Meier survival analysis revealed a significantly lower risk of progression for patients with high circHIPK3 and circCDYL expression levels compared to patients with low levels (*P* < 0.01, Log-Rank Test, Fig. 6a). In contrast, neither the expression of the corresponding linear transcript nor the host gene’s total mRNA expression correlated with BC progression after multiple testing correction (FDR > 0.1, Log-Rank Test with BH correction, Supplementary Fig. 8a–d). In agreement with the Kaplan–Meier survival analysis, circHIPK3 and circCDYL were upregulated in class 1 compared to the more aggressive BC subclasses, class 2 and 3, and more expressed in the circular than linear form (Fig. 6b). Correspondingly, both circRNA candidates were found at higher levels in non-malignant BC cell lines than metastatic BC cell lines (Fig. 6c). For both candidates, we evaluated the expression levels based on either Poly(A) selected RNA-Seq or total RNA in three fractionated cell lines (FL3, HCV29, and T24). In total RNA fractions, we observed that circHIPK3 were localized in the cytoplasm of FL3, T24, and HCV29 cells (Fig. 6d, Supplementary Fig. 9a, b) and circCDYL were localized in the cytoplasm of FL3 and T24 cells (Fig. 6d, Supplementary Fig. 9a). CircCDYL was not detected in fractionated HCV29 cells (Supplementary Fig. 9b). As expected, neither of the candidates were detected upon Poly(A) selection (Fig. 6d, Supplementary Fig. 9a, b).

Taken together, these results indicate that circHIPK3 and circCDYL possess prognostic biomarker potential for early-stage BC.

## Discussion

In this study, we have described the overall landscape of circRNAs in NMIBC, characterized the biological features of highly expressed circRNAs, and investigated their clinical correlations. We show that a subset of highly expressed circRNAs in early-stage BC possess key biological characteristics, which distinguish them from lowly expressed circRNAs and non-circularized exons. In addition, we identify 113 circRNAs that are differentially expressed between early-stage BC risk classes. In general, we see that circRNAs are downregulated in aggressive NMIBC compared to non-aggressive NMIBC. Most importantly, we identified 13 circRNAs that are associated with BC progression independently of the linear transcript and parent gene.

We identified more than 38,000 unique circRNAs across a large cohort of 457 NMIBC samples. Consistent with previous findings in other tissues,^2,8^ most circRNAs in BC are spliced from protein-coding genes that may give rise to multiple circRNA isoforms. The genomic origins of splice sites were significantly different between circular and linearly spliced variants, suggesting that circularization is not just a by-product of linear splicing events.

We validated a handful of circRNAs by Sanger sequencing across backsplice junctions. Yet, backsplice sequences could be produced by other mechanisms such as reverse transcriptase template switching and trans-splicing.^43^ However, template switching is close to random and is not expected to coincide with annotated splice sites which is the case for 91,7% of detected circRNAs but not a prerequisite for find_circ identification, and back-splice spanning reads are reduced substantially in PolyA-enriched samples, suggesting that trans-splicing is a rare event. Jeck et al. discuss several approaches that distinguish non-circular RNA species from true circles.^43^ Among other things, they suggest that circRNAs can be validated by RNase R exonuclease treatment that degrade linear RNA and northern blot analysis. Overall, we believe that the vast majority of abundant circRNAs are true circles as they are supported by a high number of reads. Additionally, the circRNAs we highlight like ciRS-7, circCDYL, and circHIPK3 have been validated in other studies.^44–46^

We show that abundant circRNAs are more likely to possess highly conserved core splice sites, and in agreement with other studies, are more likely to be associated with inverted homologous Alu repeats.^6^ Highly conserved core splice sites could indicate an evolutionary maintenance of backsplicing. In addition, enrichment of SCEs and miRNA target sites both indicate functions of the circular sequence in addition to coding of protein.

We performed cell fractionation analysis of circularized RNAs in three cell lines. In agreement with previous findings, we observe that circRNAs are mostly found in cytoplasm.^6,15^ This localization corresponds with the most studied function of circRNAs; as miRNA sponges. However, as proposed by other studies, our analysis indicates that circRNAs do not generally function as miRNA-sponges.^47,48^ We identify only two circRNAs with repeated binding sites for a particular miRNA; ciRS-7 and a circRNA overlapping *RP11-255H23.2*. CiRS-7 is known to harbour more than 70 putative miR-7 binding sites.^7^ It has been shown that ciRS-7 is highly expressed in brain tissue where it strongly suppresses miR-7 activity.^7^ Though ciRS-7 is the circRNA with highest binding frequency of the same miRNA, we only observe 8 miR-7 Argonaute (AGO)-associated target sites in brain tissue, indicating that all miR-7 target sites are not bound by AGO, at least in analyzed tissues. Similar findings were observed by Guo et al.^47^ An alternative explanation could be that this method is not sensitive enough to capture all miRNA-target interactions. All in all, our results do not support a scenario where sponging is a canonical function of circRNAs. Noteworthy, we discover that ciRS-7 is upregulated in class 2 (high-risk) tumors. As miR-7 has been proposed a tumor-suppressive role in cancer,^49–53^ an elevated level of ciRS-7 in aggressive BC seems plausible, as sponging miR-7 renders its oncogenic targets susceptible to translation. An elevated expression level in aggressive BC is in agreement with the findings in a recent study of ciRS-7 and its prognostic biomarker potential in colorectal cancer.^54^

Accumulating evidence relates circRNAs to carcinogenesis and cancer progression.^10,28,55,56^ Their stable circular structure and the detection of circRNAs at high levels in human serum,^16–18^ adds to their biomarker potential. Especially circRNAs that can distinguish low-risk patients from high-risk patients at an early tumor stage could be clinically cost effective and treatment effective. A recent study has explored the expression of circRNAs in six bladder carcinoma tissues and paired adjacent noncancerous tissues, and in particular circTCF25’s regulation of cancer-related pathways through miRNA interactions.^57^ We only observed this circRNA species in one sample supported by two reads. However, Zhong et al. mostly analyzed MIBC tumors while our study focused on NMIBC tumors.

We highlighted circHIPK3 and circCDYL as important candidates because they possess strong clinical and biological associations. Zheng et al. suggest that circHIPK3 promotes proliferation by sponging miR-124, a reported tumor suppressor,^58–60^ and that silencing of circHIPK3 inhibits cell growth.^8^ We find only one target site for miR-124 and though we observe more miRNA target sites in circHIPK3 than expected, we do not identify single miRNAs with high binding frequency, arguing against the sponge-hypothesis. In contrast to the findings by Zheng et al., we observe that circHIPK3 (and circCDYL) are upregulated in class 1 compared to the more aggressive NMIBC classes. Correspondingly, Kaplan–Meier survival analysis revealed that patients with high expression levels of circHIPK3 and circCDYL have lower risk of progression. Neither the linear transcript nor the genes’ total mRNA expression could distinguish the low risk patients from high risk patients. It should be noted that only 31 of 457 BC patients showed progression to MIBC, decreasing the power of the statistical tests. Additionally, the median expression is not necessarily the best threshold when dividing patients into low-risk and high-risk groups. An optimal cutoff could be identified for validation in other cohorts.

In agreement with our findings, Li et al. recently reported that circHIPK3 is downregulated in BC tissues compared to normal bladder tissues and that overexpression of circHIPK3 inhibits invasion and migration of BC cells.^61^ In addition, they showed that circHIPK3 sponges miR-558, which subsequently downregulates the expression of heparanase. However, we do not observe miR-558 as a potential miRNA target of circHIPK3 in the data analyzed here.

We further showed that circHIPK3 and circCDYL show similar expression patterns between non-malignant and metastatic cell lines and that they are highly expressed across various tissues. Taken together, these results indicate that these two candidates may play a role in maintaining normal cell functions and a dysregulated level might be involved in tumorigenesis in BC. A high circular-to-linear ratio and enrichment of SCEs suggest that these circRNAs are not a mere by-product of the transcription rate but may be regulated on its own and possess additional regulatory functions. The fact that both circHIPK3 and circCDYL have been detected in extracellular vesicles^16,17^ adds to their potential as biomarkers. However, it remains unclear whether circHIPK3 and circCDYL add prognostic value to the currently used clinical indicators. Future studies should address whether circRNAs that correlate with BC progression are present in human urine samples, and importantly, validation in independent cohorts should be performed in order to confirm their clinical relevance. Additionally, a multivariate analysis would be needed to determine whether circRNA candidates present independent prognostic value.

In summary, we have profiled the expression of circRNAs in early-stage BC and showed that abundant circRNAs have conserved core splice sites, which are likely to be surrounded by inverted homologous Alu pairs, and are enriched with SCEs and miRNA target sites. In addition, differential expression analysis and clinical correlations revealed circRNAs that are dysregulated between prognostic NMIBC classes and are associated with progression. Overall, we have shown that circRNAs might represent a new class of prognostic biomarkers in NMIBC. In particular, we point to circHIPK3 and circCDYL as they appear to retain biological and clinical value.

## Material and methods

### Detection and annotation of circRNAs

The 457 NMIBC samples were whole transcriptome sequenced and mapped in a previous study.^29^ We used the *find_circ* pipeline^2^ to find the circular RNAs but increased stringency by filtering the mapping qualities (cut-off value 35) for both anchor sequences as suggested by Venø et al.^30^

The same pipeline was used to identify circRNAs in 113 publicly available tissue samples obtained from ENCODE (Supplementary Methods).

### Genomic annotation

Linear and circular spliced transcripts supported by more than two reads in at least two different samples were analyzed for their overlap with functional regions in the genome. The functional regions were defined based on GENCODE v. 19 transcripts. Bases were hierarchically divided into coding sequences (CDS), 5′-UTR, 3′-UTR, and non-coding RNA regions (ncRNA). We obtained exon positions by using exon annotations from RefSeq database downloaded from the UCSC Genome Browser (hg19) (https://genome.ucsc.edu/).^62^ Exons were then intersected and annotated with the functional regions. To assign genomic origin to splice sites that give rise to circular and linear transcripts, we intersected a window of 6 bp surrounding the splice sites with the annotated exons. A preference hierarchy was applied if splice sites overlapped multiple annotations: CDS > 3′-UTR > 5′-UTR > ncRNA.

Based on the existing transcript annotations, we analyzed the number of exons within circRNAs and the total length of exonic circRNAs (circRNAs composed of exons). Overlapping exons were collapsed. Introns were assumed to be spliced out.

The circular-to-linear ratios were calculated by dividing the number of circular reads with the corresponding number of linear reads in each sample. A pseudocount of 1 was added to both measurements to avoid division by 0 ((#circular reads + 1)/(#linear reads + 1)). The linear counterpart of circRNAs were calculated as the sum of linear reads, using the same splice sites.

### Alu repeats

The Alu repeats were obtained from the UCSC Browser RepeatMasker track.^63^ Internal, spliced exons were obtained from the RefSeq Track by removing all transcripts with less than three exons followed by removal of all first and last exons.

Flanking regions (20 kb) around spliced exons and circRNAs were intersected with Alu Repeats. Inverted homologous Alu repeats in separate flanks of exons and circRNAs were annotated along with their closest distance. Alu repeats within the same subfamily, e.g., AluJ or AluS, were considered homologous.

### Conservation of core splice sites

To evaluate splice site conservation, we calculated the average Phylo-P conservation score^64^ of the two bases upstream and downstream of spliced exons and circRNAs.

### Enrichment of SCEs

SCEs identified at the 15-codon resolution were obtained from Lin et al.^31^ Using the UCSC liftOver Tool (http://genome.ucsc.edu/cgi-bin/hgLiftOver), regions were converted to the hg19 assembly.

To evaluate the fraction of exons containing SCEs, we obtained coding exons within the three data sets; abundant circRNAs, non-abundant circRNAs, and all spliced exons. First, we defined linearly spliced coding exons by requiring a minimum overlap of 90% with coding bases. Overlapping coding exons were collapsed. Next, circularized coding exons were defined as having 100% overlap to a linearly spliced coding exon. The coding exons were then hierarchically annotated, based on their circRNA overlap, such that exons within abundant circRNAs took precedence over exons within non-abundant circRNA, which took precedence over linearly spliced exons. The coding exons were then annotated with their SCE overlap.

### Observed versus expected SCEs

We analyzed the enrichment of SCEs for the abundant circRNAs by comparing the observed SCE overlap with the expected SCE overlap. The probability of SCE overlap (*P*_SCE_) per bp was obtained by dividing the total length of SCEs (*L*_SCE_) with the total length of unique abundant circularized coding exons (*L*_coding_exons_) (*P*_SCE_ = *L*_SCE_/*L*_coding_exons_). The expected length of SCE overlap per circRNA (SCE_exp_) was then calculated as the length of circularized coding exons per circRNA (L_coding_circRNA_) multiplied by the probability of SCE overlap per bp (SCE_exp_ = *P*_SCE_ × *L*_coding_circRNA_).

### Enrichment of miRNA target sites

An AGO-CLIP atlas of miRNA target sites was produced as previously described.^32,65^ Briefly, 106 individual human AGO-CLIP data sets, which to the best of our knowledge represents all publically available AGO-CLIP data at the time of atlas construction, were logically grouped into 32 independent experiments. Each experiment was then mapped to the hg19 genome and read groups were stacked into individual clusters. The genome sequence of each cluster was then analyzed for miRNA seed compliments using the TargetScan algorithm^66^ to define individual miRNA binding sites. The only modification from previous reports^65^ was that all files were processed exclusively through the Piranha 2 clustering algorithm^67^ so that cluster definition in genome space was consistent across experiments. We further restricted our analysis to conserved miRNAs as this reduces redundant calls and focuses analysis on more highly expressed miRNAs.

The fraction of coding exons containing miRNA binding sites were evaluated as described under Enrichment of SCEs. Many miRNAs have overlapping target sites. We merged these regions and identified the fraction of coding exons that is covered by miRNA target sites within the three coding exon groups defined in ‘Enrichment of SCEs’.

### Observed versus expected miRNAs

We analyzed the enrichment of miRNAs for the abundant circRNAs by comparing the observed number of miRNAs with the expected number of miRNAs. Exonic circRNAs as well as non-exonic circRNAs (circRNAs that do not overlap annotated exons) with a size of less than 10,000 bp were included in this analysis. The probability of miRNA binding site overlap (*P*_miRNA_) per nucleotide was obtained by dividing the total number of miRNAs (*T*_miRNAs_) with the total length of the circRNAs (*L*_exons_) (for exonic circRNAs introns were assumed to be spliced out) (*P*_miRNA_ = *T*_miRNAs_/*L*_exons_). The expected number of miRNAs per circRNA (miRNA_exp_) was calculated as the length of the circRNA (*L*_circRNA_) multiplied by the miRNA binding sites overlap per nucleotide (miRNA_exp_ = *P*_miRNA_ × *L*_circRNA_).

### Statistical analyses

All statistical tests were performed in R.^68,69^ The non-parametric Wilcoxon rank sum test was carried out to assess differential expression between prognostic BC risk classes. Wilcoxon rank sum test was also utilized to evaluate differential circular-to-linear expression between risk classes. Kaplan–Meier plots were produced with the “Survival”^70,71^ and “Survminer”^72^ R packages and curves were compared statistically by the log-rank test, using critical value from the *χ* ^2^ table. BH correction were applied for multiple testing correction and statistical differences were declared significant at FDR < 0.1. In the analyses where multiple testing correction was not applied, statistical differences were declared significant at *P* < 0.05.

Heatmaps were made with the R package heatmap3^73^ and most plots were produced with the R package ggplot2.^74^

### Cell lines

BC cell lines RT4, HT1376, T24 (American Type Culture Collection (ATCC)), FL3 (Prof. Dan Theodorescu, University of Colorado Cancer Center, Aurora, CO, USA) and immortalized human bladder epithelium HCV29 (Prof. Jesper Zeuthen, Danish Cancer Society, Copenhagen, Denmark)) cells were used in the present study. T24 and RT4 cells were grown in McCoy’s 5 A media (Gibco, Life Technologies, Carlsbad, CA, USA). FL3 cells were grown in DMEM/F12 media (Gibco, Life Technologies) with 2% L-glutamine (Gibco, Life Technologies) and HCV29 cells were grown in DMEM (Gibco, Life Technologies). Finally, HT1376 cells were propagated in MEM media (Gibco, Life Technologies) with 1% non-essential amino acids (Gibco, Life Technologies) and 2% L-Glutamine (Gibco, Life Technologies). The culture media was supplemented with 10% Fetal Calf Serum (FCS) (Gibco, Life Technologies) and 1% Penicillin-Streptomycin (Gibco, Life Technologies). Cells were cultured at 37 °C in an atmosphere of 5% CO_2_. Cell line authentication was confirmed using Cell-ID (Promega, Fitchburg, WI, USA).

### CircRNA profiling in unfractionated and fractionated cell lines

Whole transcriptome RNA-Seq data of unfractionated RNA from eight bladder cancer cell lines (Nhutert, HCV29, HT1197, RT4, J82, HT-1376, T24, and UMUC3) were generated and mapped in a previous study.^29^

The Protein And RNA Isolation System (PARIS) (Ambion, Life Technologies, Foster City, CA, USA) was used to partition FL3, HCV29 and T24 cells into cytoplasmic and nuclear fractions followed by isolation of RNA from each fraction. Briefly, 6 × 10^6^ cells were harvested and re-suspended in cell fractionation buffer followed by low speed centrifugation (500x*g*) at 4 °C for 5 min. Subsequently, the RNA was isolated from the supernatant (cytoplasmic fraction) and the pellet (whole nuclei) according to the manufacturer’s instructions. Whole transciptome and strand-specific RNA-Seq data from the fractionated cells were prepared using the Ribo-Zero technology (Epicentre, an Illumina company) for depletion of rRNA followed by library preparation using the ScriptSeq technology (Epicentre, an Illumina company). RNA-Seq of the poly(A) transcripts from the fractionated cells were prepared using the Dynabeads mRNA DIRECT Micro Kit (Ambion, Life Technologies) followed by library preparation using the ScriptSeq technology (Epicentre, an Illumina company). Input for both Ribo-Zero and poly(A) selection were 3 µg RNA from each of the cytoplasmic fractions while 500 ng RNA was used from the nucleic fractions. The nucleic and cytoplasmic libraries were 2 × 75 bp sequenced on a NextSeq 500 system (Illumina).

The raw reads were converted to fastq format and demultiplexed using Illumina’s bcl2fastq v2.18.0.12 and library adapters were removed from the read pairs (trim_galore v0.4.1). Reads were mapped to the human genome (hg19) using TopHat2 (version 2.1.1)^75^ and Bowtie2 (version 2.1.0.0)^76^ and Cufflinks (v2.1.1)^77^ and HTSeq (v0.6.1p1)^78^ were used to estimate the transcripts abundance using transcript information from GENCODE v19. Samtools (v1.3)^79^ and Picard (v2.0.1) were used for quality control and statistics.

*Find_circ* pipeline^2^ with our increased filtering stringency was used to detect the circRNAs in RNA-Seq data from unfractionated cells and from the nucleic and cytoplasmic fractions. The data have been deposited in NCBI’s Gene Expression Omnibus^80^ and are accessible through GEO Series accession number GSE100971 (https://www.ncbi.nlm.nih.gov/geo/query/acc.cgi?acc=GSE100971).

### Validation of selected circRNAs

Primers aligning divergently at the genomic level in the implicated exons of the respective cirRNAs were used to verify the presence of the exon backsplicing sites of circHIPK3, circFNDC3B, circPCMTD1, circZNF609, and circZMYM4 in bladder cell lines (primer sequences are shown in Supplementary Table 5). Initially, total RNA was isolated from T24, FL3, HCV29, RT4 and HT1376 cells using RNeasy Mini Kit (Qiagen, Hilden, Germany) following the manufacturer’s instructions. Subsequently, first strand cDNA was synthesized using 1 µg of RNA and SuperScript II Reverse Transcriptase (Invitrogen, ThermoFisher Scientific, Carlsbad CA, USA) as described by the manufacturer. The generated cDNA was used as template in a temperature-gradient PCR with the divergent primers using the following PCR profile: 95 °C for 3 min followed by 30 cycles of denaturation at 95 °C for 15 s, annealing at 52–68 °C for 30 s and synthesis at 72 °C for 1 min, and finally 72 °C for 3 min and on hold at 15 °C. The PCR products were run on a 2% agarose gel to verify the presence of a single PCR product of correct size. The circHIPK3 was detected in T24, FL3, HCV29 cells, circFNDC3B in HT1376, whereas circZNF609, circPCMTD1 and circZMYM4 were detected in RT4 cells. Subsequently, gel fragments containing the PCR products were cut out of the gel and the DNA was purified using the GFX PCR DNA and Gel Purification Kit (GE Healthcare) following manufacturer’s instructions and eluted in 20 µl H_2_0. The purified PCR products were cloned into the pCR™4-TOPO^®^ vector and transformed into one Shot^®^ TOPO10 E. coli cells using the TOPO^®^ TA Cloning^®^ Kit for Sequencing (Invitrogen, ThermoFisher Scientific) as indicated by the manufacturer. Thirty µl of the transformation mix was plated out on a pre-warmed ampicillin-resistance-selective agar plate and incubated overnight at 37 °C. To verify the cloning of the PCR products, colony PCR was carried out using M13 forward (5′-GTAAAACGACGGCCAG-3′) and M13 reverse primers (5′-CAGGAAACAGCTATGAC-3′). The colonies from the agar plates were used directly as templates in the PCR. In all, five colonies were tested from each plate. The following PCR conditions were used: 95 °C for 10 min followed by 26 cycles of denaturation at 95 °C for 30 s, annealing at 56 °C for 30 s and synthesis at 72 °C for 1 min and finally 1 cycle of 72 °C for 1 min and on hold at 10 °C. Finally, GenElute™ Plasmid Miniprep Kit (Sigma Aldrich, Merck, Darmstadt, Germany) was applied to isolate plasmid DNA from colonies that were tested positive in the colony PCR. Sanger sequencing with BigDye^®^ Terminator v1.1 Cycle Sequencing Kit (ThermoFisher Scientific) was carried out according to the manufacturer’s instructions with few modifications to verify the sequences of the cloned PCR products. Briefly, 2 µl BigDye™ Terminator v1.1 Ready Reaction Mix, 1 µl primer (M13 forward or M13 reverse.; 2 pmol/l), 1.5 µl BigDye™ Terminator v1.1 & v3.1 5× Sequencing Buffer, 4.5 l H_2_O (Accugen) and 1 µl template (plasmid DNA) were used in the cycle sequencing reaction. The cycle sequencing reaction was performed on a Bio-Rad C1000 ThermalCycler (Bio-Rad, Basel, Switzerland) using the following conditions: 96 °C for 1 min followed by 26 cycles of denaturation at 96 °C for 10 s, annealing at 50 °C for 5 s and synthesis at 60 °C for 4 min. The product of the cycle sequencing reaction was purified with the CLEANSEQ^®^ Dye-Terminator Removal kit (AGENCOURT^®^) (Beckman Coulter, Brea, CA, USA) on a Biomek NXp (Beckman Coulter) and run on a ABI 3500XL3 Genetic Analyzer (Applied Biosystems, Life Technologies, Carlsbad, CA, USA). Generated DNA sequences were aligned to relevant circRNA sequences from the genome ((human hg19 (February 2009, GRCh37)) using Sequencher v5.4.5 (Gene Codes, Ann Arbor, MI, USA).

### Data availability

The patient sequencing data that support the findings of this study are available at the European Genome-Phenome Archive under accession number EGAS00001001236. The tissue sequencing data that support the findings of this study are available from ENCODE, https://www.encodeproject.org/. The cell line and fractionated cell line sequencing data have been deposited in NCBI’s Gene Expression Omnibus and are accessible through GEO Series accession number GSE100971, https://www.ncbi.nlm.nih.gov/geo/query/acc.cgi?acc = GSE100971. All other relevant data are available from the corresponding author on request.

## Electronic supplementary material

Supplementary Note

Supplementary Figures

Supplementary Table 1

Supplementary Table 2

Supplementary Table 3

Supplementary Table 4

Supplementary Table 5

Supplementary Methods
