# Supplementary material for: Circular RNA expression is abundant and correlated to aggressiveness in early-stage bladder cancer
Source: NPJ Genom Med. 2017 Nov 28;2:36. doi: 10.1038/s41525-017-0038-z (PMC5705701; doi:10.1038/s41525-017-0038-z)
Supplement: Supplementary file 1 — Supplementary Note [file 41525_2017_38_MOESM1_ESM.docx]

**Supplementary Figure Legends**

**Supplementary Figure 1. Validation of circRNAs.** Sanger sequencing across backsplice junction of **a** circHIPK3, **b** circFNDC3B, **c** circPCMTD1, **d** circZNF609, and **e** circZMYM4. The sequence across the backsplice-junction is highlighted.

**Supplementary Figure 2. Statistical analyses supporting Figure 2. a** Distance between the closest pair of inverted homologous Alu repeats surrounding abundant circRNAs, non-abundant circRNAs, and linearly spliced exons within a window of 20 kb, omitting the size of the circRNAs or spliced exons themselves. If there is no inverted homologous Alu repeats within the interrogated window, the distance have been set to 22,000 kb in order to include them in the statistical analysis. **P<0.01, ***P<0.001 (Wilcoxon Rank Sum Test, black dots represent mean). **b** The average PhyloP position-specific conservation score of the four nucleotides involved in splicing. ***P<0.001, ns = not significant (Wilcoxon Rank Sum Test, black dots represent mean). **c** Fraction of coding exons overlapped by SCEs (abundant vs non-abundant circRNAs, P < 0.01; non-abundant circRNAs vs linear exons, P < 0.001; abundant circRNAs vs linear exons, P < 0.001, Kolmogorov-Smirnov Test). **d** Coding exon size (ns = not significant, Wilcoxon Rank Sum Test, black dots represent mean). **e** SCE coverage per nucleotide (***P < 0.001, Wilcoxon Rank Sum Test, black dots represent mean). **f** Fraction of coding exons overlapped by miRNA binding site regions (abundant vs non-abundant circRNAs, P-value not significant; non-abundant circRNAs vs linear exons, P < 0.001; abundant circRNAs vs linear exons, P < 0.001, Kolmogorov-Smirnov test). **g** miRNA binding sites per nucleotide (*P < 0.05, ***P<0.001, Wilcoxon Rank Sum Test, black dots represent mean).

**Supplementary Figure 3. Correlation between circRNA expression and ADAR and Quaking expression. a+b** Correlation of total circular expression per sample and **a** *Quaking* (QKI) expression per sample (Spearman’s rho = 0.323, P-value = 1.86e-12) **b** *ADAR* expression per sample (Spearman’s rho = 0.0568, P-value = 0.226). **c+d** Spearman Correlation between 279 abundant circRNAs and **c** *Quaking* expression **d** *ADAR* expression.

**Supplementary Figure 4. Expression of abundant circRNAs in the fractionated bladder cancer cell lines T24 and HCV29.** Circular expression of abundant circRNAs in the cytoplasmic and nuclear cellular compartments of **a** T24 (n = 133) and **b** HCV29 (n = 93).

**Supplementary Figure 5. Expression of abundant circRNAs across 113 tissue samples.**

**Supplementary Figure 6. Heatmap of 279 abundant circRNAs in 457 NMIBC samples with clinical and biological annotations.** Heatmap of 279 abundant circRNAs. Expression levels in counts per million (CPM) are denoted with a colour gradient (right part). White denotes no reads. Samples are ordered according to their EORTC score. For each sample, coloured bars above expression heatmap denote stage, grade, prognostic class, library size and EORTC score and whether or not it progresses to muscle-invasive bladder cancer (T2+). Properties of individual circRNAs are denoted with larger coloured bars (left of expression heatmap). Properties that can be represented by Yes/No/NA: *iii*) Known cancer gene (does the circRNA arise from a known cancer gene); *iv*) Circ-to-lin ratio > 1 (is the average circular-to-linear expression level across all samples greater than 1); *v*) SCE, obs > exp (is the observed SCE coverage higher than expected SCE coverage based on exon size); *vi*) miRNA, obs > exp (is the observed miRNA coverage higher than expected miRNA coverage based on exon size); *vii*) ADAR correlation (is circRNA expression correlated with ADAR expression, Spearman correlation); *viii*) Quaking correlation (is circRNA expression correlated with Quaking expression, Spearman correlation); *ix*) Cell lines (are the circRNAs detected in at least one bladder cancer cell line);  *x*) EORTC correlation (is circRNA expression correlated with EORTC score, Spearman correlation); *xi*) Diff. exp., class 1 vs 2 (differential expression analysis between class 1 and 2, Wilcoxon Rank Sum Test);  *xii*) circ-to-lin diff. exp., class 1 vs 2 (differential expression analysis of the circular-to-linear ratio between class 1 and 2); *xiii*) KM circ (Kaplan-Meier analysis of the circular transcript), *xiv*) KM lin (Kaplan-Meier analysis of the corresponding linear transcript); *xv*) KM mRNA (Kaplan-Meier analysis of the parent gene (mRNA)). The Benjamini Hochberg procedure were used for multiple testing correction and FDR < 0.1 were declared significant.

Properties that range high/far to low/near: *i*) Conservation core splice sites (average conservation of the four core nucleotides involved in splicing); *ii*) Alu repeats (the distance between the pair of inverted homologous Alu repeats in closest proximity with the circle); *xvi*) No. of tissues (in how many of the 113 tissues is the circRNA expressed).

*xvii*) Expression levels in CPM in bladder cancer tissue are denoted with the same colour gradient as the heatmap.

**Supplementary Figure 7.** **Correlation between circRNAs and the EORTC score.** Spearman Correlation between 279 abundant circRNAs and the EORTC score.

**Supplementary Figure 8. Kaplan Meier plots of the linear counterpart and parent gene of circHIPK3 and circCDYL.** Kaplan-Meier progression plots of **a** the linear counterpart of circHIPK3, **b** the gene *HIPK3,* **c** the linear counterpart of circCDYL, and **d** the gene *CDYL*. Median expression used as cutoff. P-values obtained by log-rank test.

**Supplementary Figure 9. Fractionated cell line expression.** Circular and linear expression of HIPK3 and CDYL in the cytoplasm and nucleus of **a** T24 and **b** HCV29. Expression levels are measures based on either poly(A) selected RNAseq (PolyA; only mRNAs) or totalRNA (both mRNAs and circRNAs).

**Supplementary Tables**

**Supplementary Table 1: Clinical annotations of 457 NMIBC samples.**

**Supplementary Table 2: Genomic location and annotations from differential expression analyses of 279 abundant circRNAs.**

**Supplementary Table 3: Target sites of the miRNAs that bind at highest frequency for ciRS-7 and circRP11-255H23.2.**

**Supplementary Table 4: Summary of clinical and biological features of 13 circRNAs that independently correlate with BC progression.**

**Supplementary Table 5: Primer sequences for validated circRNAs.**

**Supplementary Methods**

**Supplementary Methods: Bash script containing the code to extract 113 tissue files from ENCODE.**
