# Supplementary material for: Circular RNA expression is abundant and correlated to aggressiveness in early-stage bladder cancer
Source: NPJ Genom Med. 2017 Nov 28;2:36. doi: 10.1038/s41525-017-0038-z (PMC5705701; doi:10.1038/s41525-017-0038-z)
Supplement: Supplementary file 2 — Supplementary Figures [file 41525_2017_38_MOESM2_ESM.pdf]

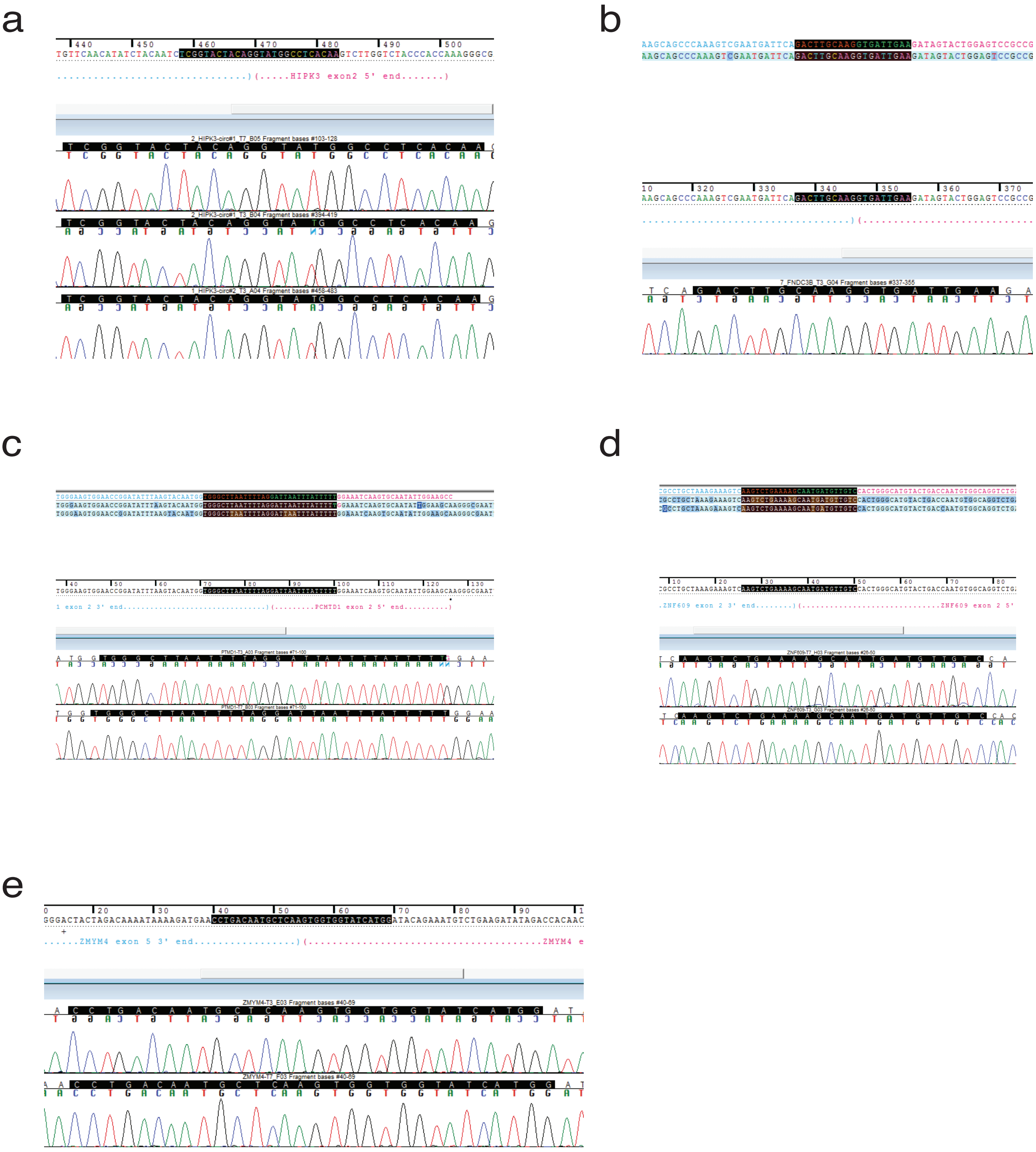

Supplementary Figure 1. Validation of circRNAs. Sanger sequencing across backsplice junction of **a** circHIPK3, **b** circFNDC3B, **c** circPCMTD1, **d** circZNF609, and e circZMYM4. The sequence across the backsplice-junction is highlighted.

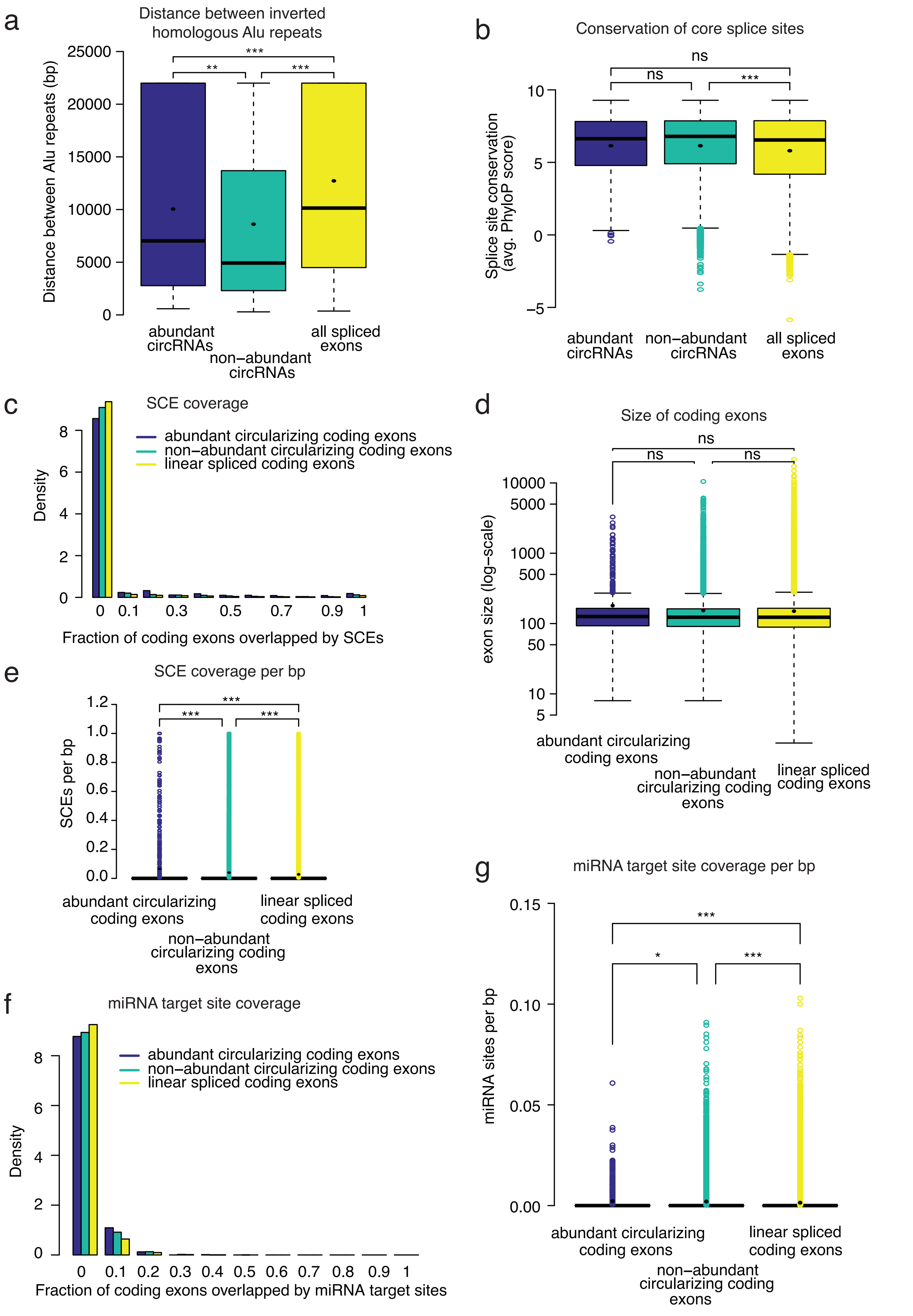
a
Distance between inverted homologous Alu repeats
Distance between Alu repeats (bp)
25000
20000
15000
10000
5000
0
***
**
***
abundant circRNAs
non−abundant circRNAs
all spliced exons
b
Conservation of core splice sites
Splice site conservation (avg. PhyloP score)
10
5
0
−5
ns
ns
***
abundant circRNAs
non−abundant circRNAs
all spliced exons
c
SCE coverage
abundant circularizing coding exons
non−abundant circularizing coding exons
linear spliced coding exons
Density
8
6
4
2
0
0 0.1 0.3 0.5 0.7 0.9 1
Fraction of coding exons overlapped by SCEs
d
Size of coding exons
exon size (log−scale)
10000
5000
1000
500
100
50
10
5
ns
ns
ns
abundant circularizing coding exons
non−abundant circularizing coding exons
linear spliced coding exons
e
SCE coverage per bp
SCEs per bp
1.2
1.0
0.8
0.6
0.4
0.2
0.0
***
***
***
abundant circularizing coding exons
non−abundant circularizing coding exons
linear spliced coding exons
f
miRNA target site coverage
abundant circularizing coding exons
non−abundant circularizing coding exons
linear spliced coding exons
Density
8
6
4
2
0
0 0.1 0.2 0.3 0.4 0.5 0.6 0.7 0.8 0.9 1
Fraction of coding exons overlapped by miRNA target sites
g
miRNA target site coverage per bp
miRNA sites per bp
0.15
0.10
0.05
0.00
***
*
***
abundant circularizing coding exons
non−abundant circularizing coding exons
linear spliced coding exons

Supplementary Figure 2. Statistical analyses supporting Figure 2. a Distance between the closest pair of inverted homologous Alu repeats surrounding abundant circRNAs, non-abundant circRNAs, and linearly spliced exons within a window of 20 kb, omitting the size of the circRNAs or spliced exons themselves. If there is no inverted homologous Alu repeats within the interrogated window, the distance have been set to 22,000 kb in order to include them in the statistical analysis. **P<0.01, ***P<0.001 (Wilcoxon Rank Sum Test, black dots represent mean). **b** The average PhyloP position-specific conservation score of the four nucleotides involved in splicing. ***P<0.001, ns = not significant (Wilcoxon Rank Sum Test, black dots represent mean). **c** Fraction of coding exons overlapped by SCEs (abundant vs non-abundant circRNAs, P < 0.01; non-abundant circRNAs vs linear exons, P < 0.001; abundant circRNAs vs linear exons, P < 0.001, Kolmogorov-Smirnov Test). **d** Coding exon size (ns = not significant, Wilcoxon Rank Sum Test, black dots represent mean). **e** SCE coverage per nucleotide (***P < 0.001, Wilcoxon Rank Sum Test, black dots represent mean). **f** Fraction of coding exons overlapped by miRNA binding site regions (abundant vs non-abundant circRNAs, P-value not significant; non-abundant circRNAs vs linear exons, P < 0.001; abundant circRNAs vs linear exons, P < 0.001, Kolmogorov-Smirnov test). **g** miRNA binding sites per nucleotide (*P < 0.05, ***P<0.001, Wilcoxon Rank Sum Test, black dots represent mean).

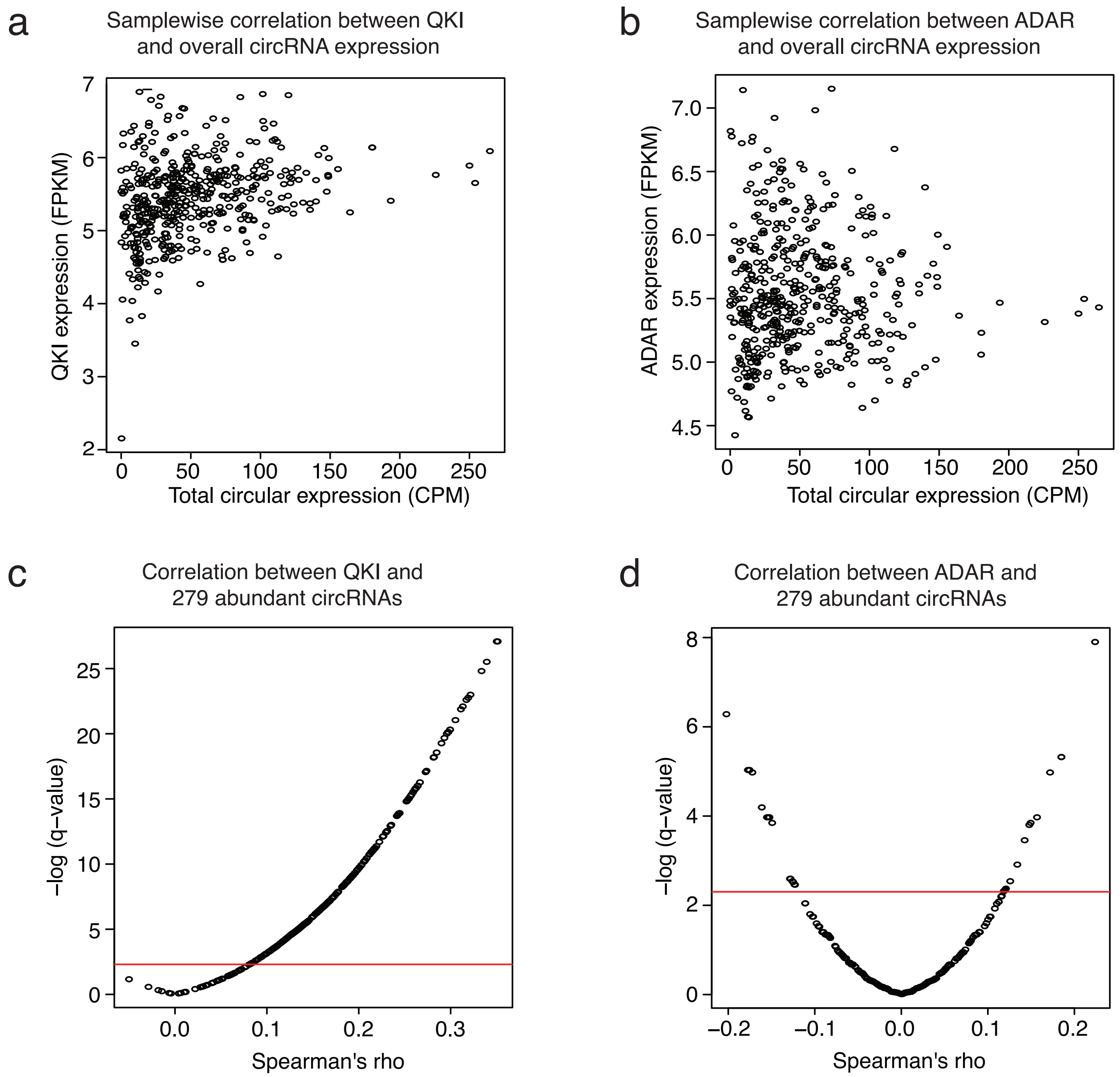

Supplementary Figure 3. Correlation between circRNA expression and ADAR and Quaking expression. a+b Correlation of total circular expression per sample and **a** Quaking (QKI) expression per sample (Spearman's rho = 0.323, P-value = 1.86e-12) **b** ADAR expression per sample (Spearman's rho = 0.0568, P-value = 0.226). **c+d** Spearman Correlation between 279 abundant circRNAs and **c** Quaking expression **d** ADAR expression.

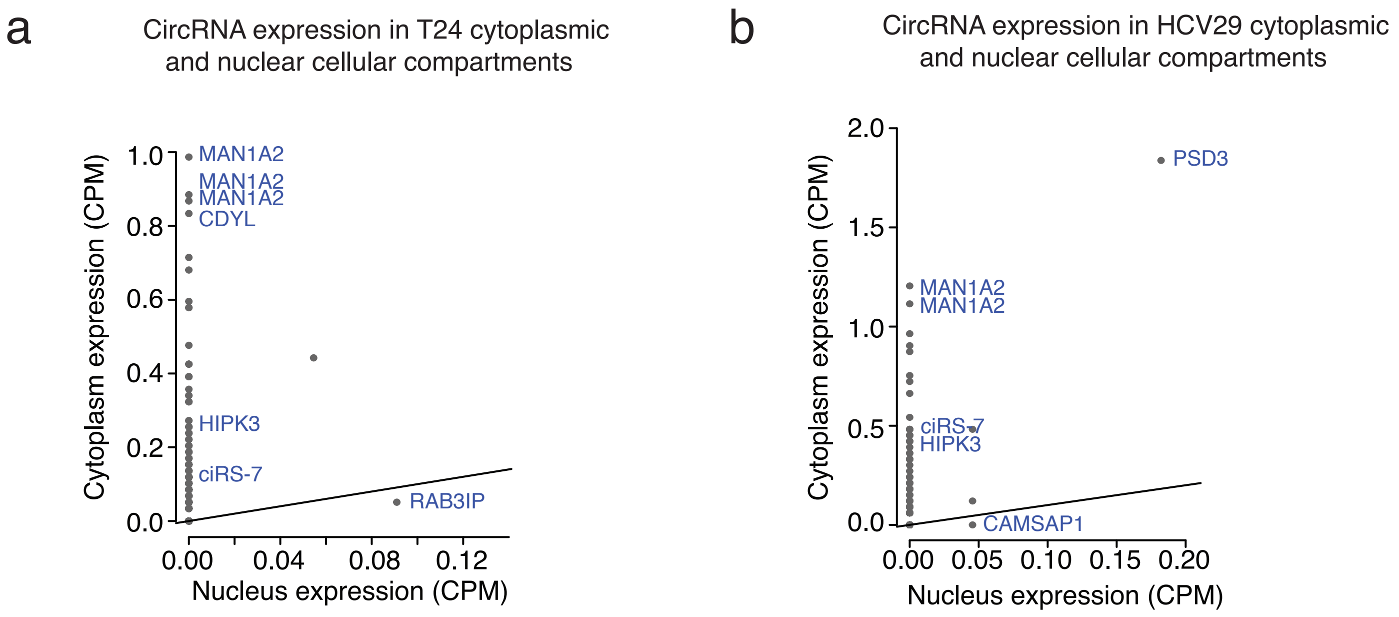

Supplementary Figure 4. Expression of abundant circRNAs in the fractionated bladder cancer cell lines T24 and HCV29. Circular expression of abundant circRNAs in the cytoplasmic and nuclear cellular compartments of **a** T24 (n = 133) and **b** HCV29 (n = 93).

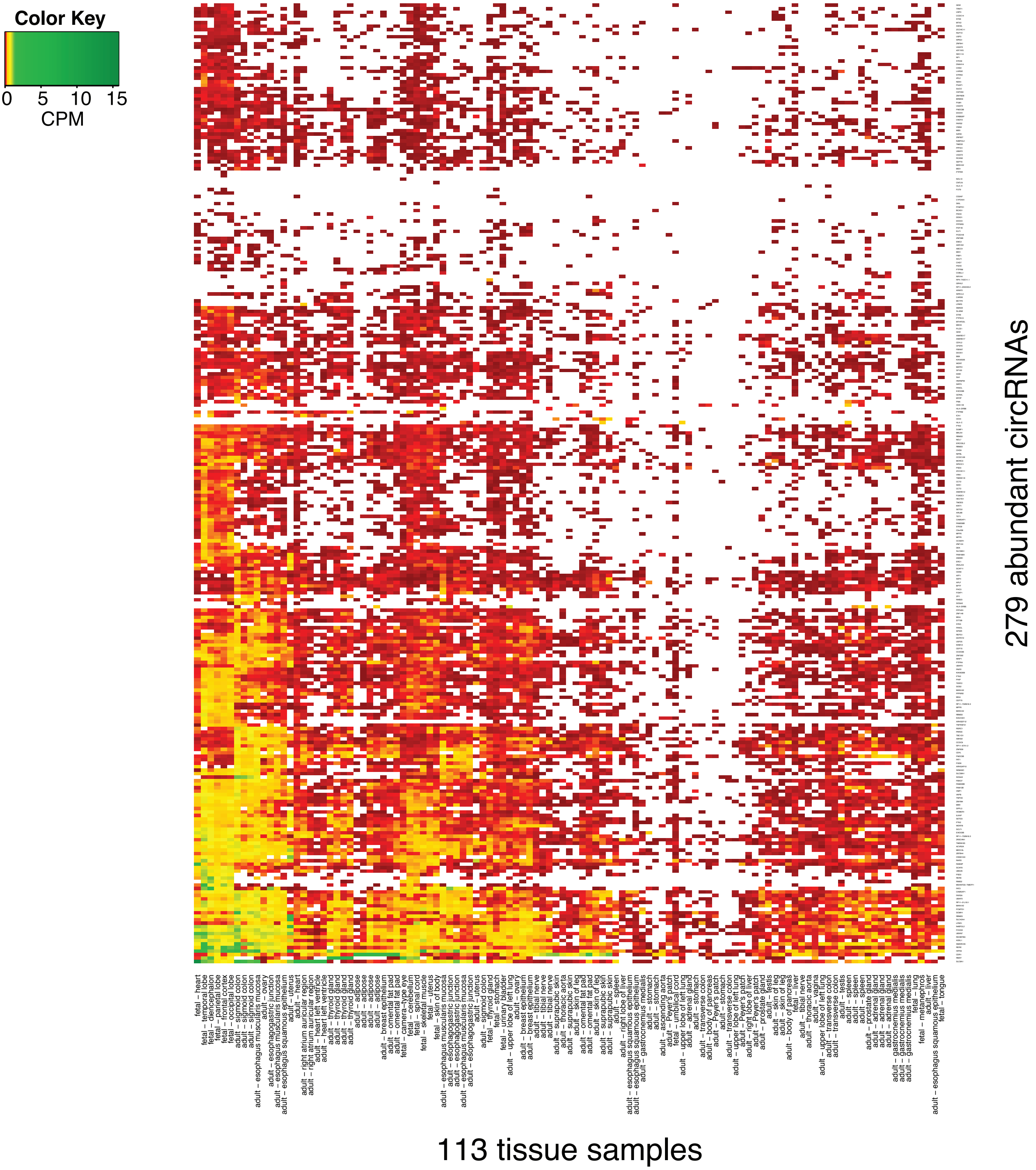

Supplementary Figure 5. Expression of abundant circRNAs across 113 tissue samples.

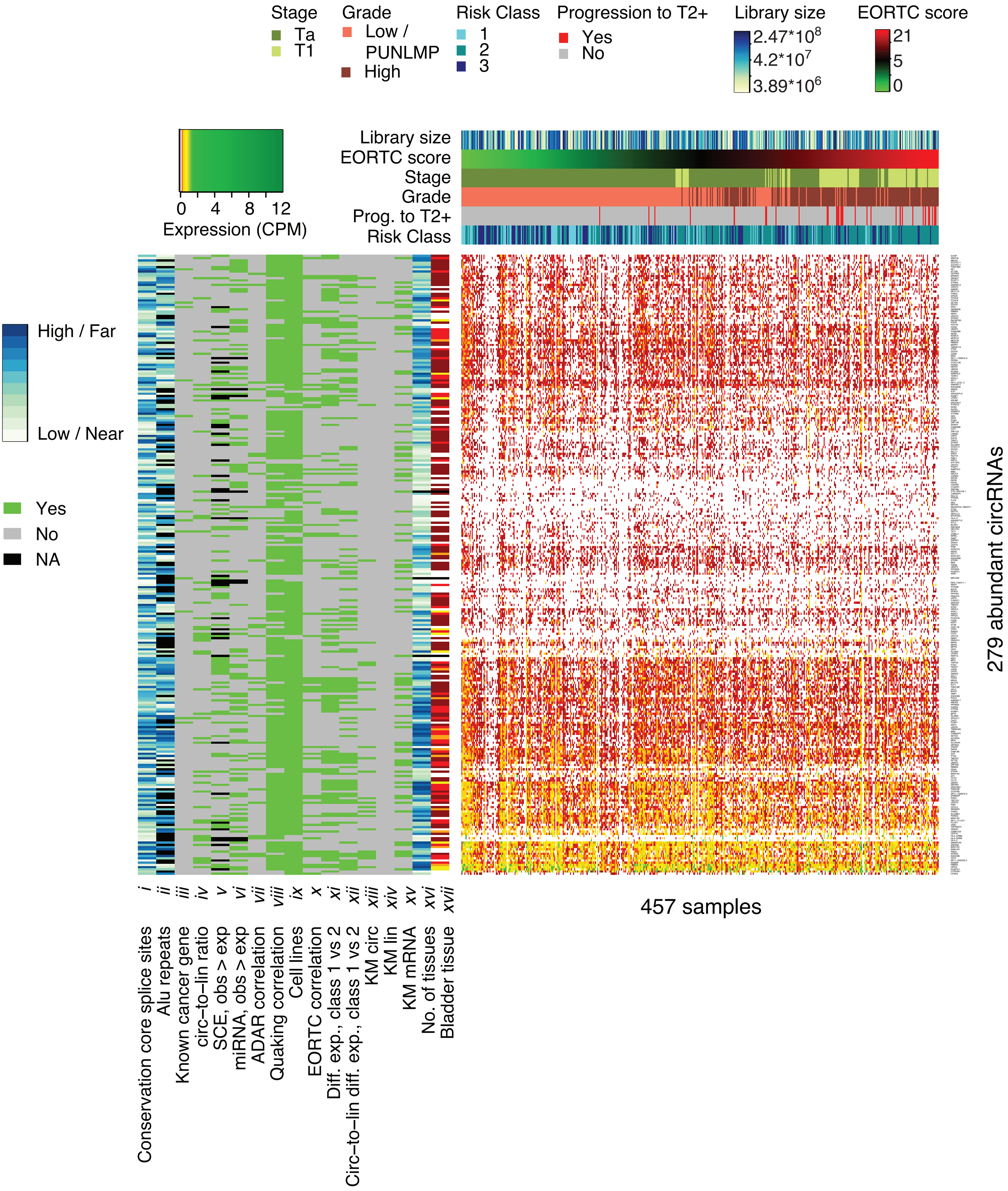

Stage
Ta
T1
Grade
Low / PUNLMP
High
Risk Class
1
2
3
Progression to T2+
Yes
No
Library size
2.47*10^8
4.2*10^7
3.89*10^6
EORTC score
21
5
0
0 4 8 12
Expression (CPM)
Library size
EORTC score
Stage
Grade
Prog. to T2+
Risk Class
High / Far
Low / Near
Yes
No
NA
279 abundant circRNAs
457 samples
i Conservation core splice sites
ii Alu repeats
iii Known cancer gene
iv circ−to−lin ratio
v SCE, obs > exp
vi miRNA, obs > exp
vii ADAR correlation
viii Quaking correlation
ix Cell lines
x EORTC correlation
xi Diff. exp., class 1 vs 2
xii Circ−to−lin diff. exp., class 1 vs 2
xiii KM circ
xiv KM lin
xv KM mRNA
xvi No. of tissues
xvii Bladder tissue

Supplementary Figure 6. Heatmap of 279 abundant circRNAs in 457 NMIBC samples with clinical and biological annotations. Heatmap of 279 abundant circRNAs. Expression levels in counts per million (CPM) are denoted with a colour gradient (right part). White denotes no reads. Samples are ordered according to their EORTC score. For each sample, coloured bars above expression heatmap denote stage, grade, prognostic class, library size and EORTC score and whether or not it progresses to muscle-invasive bladder cancer (T2+). Properties of individual circRNAs are denoted with larger coloured bars (left of expression heatmap). Properties that can be represented by Yes/No/NA: iii) Known cancer gene (does the circRNA arise from a known cancer gene); iv) Circ-to-lin ratio > 1 (is the average circular-to-linear expression level across all samples greater than 1); v) SCE, obs > exp (is the observed SCE coverage higher than expected SCE coverage based on exon size); vi) miRNA, obs > exp (is the observed miRNA coverage higher than expected miRNA coverage based on exon size); vii) ADAR correlation (is circRNA expression correlated with ADAR expression, Spearman correlation); viii) Quaking correlation (is circRNA expression correlated with Quaking expression, Spearman correlation); ix) Cell lines (are the circRNAs detected in at least one bladder cancer cell line); x) EORTC correlation (is circRNA expression correlated with EORTC score, Spearman correlation); xi) Diff. exp., class 1 vs 2 (differential expression analysis between class 1 and 2, Wilcoxon Rank Sum Test); xii) circ-to-lin diff. exp., class 1 vs 2 (differential expression analysis of the circular-to-linear ratio between class 1 and 2); xiii) KM circ (Kaplan-Meier analysis of the circular transcript), xiv) KM lin (Kaplan-Meier analysis of the corresponding linear transcript); xv) KM mRNA (Kaplan-Meier analysis of the parent gene (mRNA)). The Benjamini Hochberg procedure were used for multiple testing correction and FDR < 0.1 were declared significant.
Properties that range high/far to low/near: i) Conservation core splice sites (average conservation of the four core nucleotides involved in splicing); ii) Alu repeats (the distance between the pair of inverted homologous Alu repeats in closest proximity with the circle); xvi) No. of tissues (in how many of the 113 tissues is the circRNA expressed).
xvii) Expression levels in CPM in bladder cancer tissue are denoted with the same colour gradient as the heatmap.

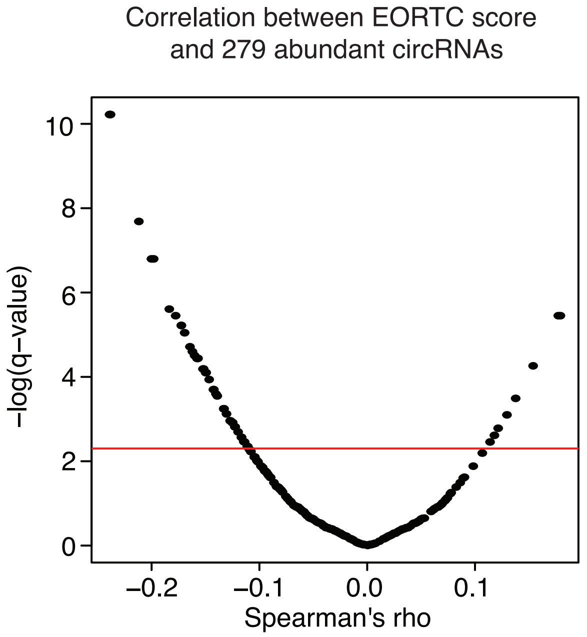

Supplementary Figure 7. Correlation between circRNAs and the EORTC score. Spearman Correlation between 279 abundant circRNAs and the EORTC score.

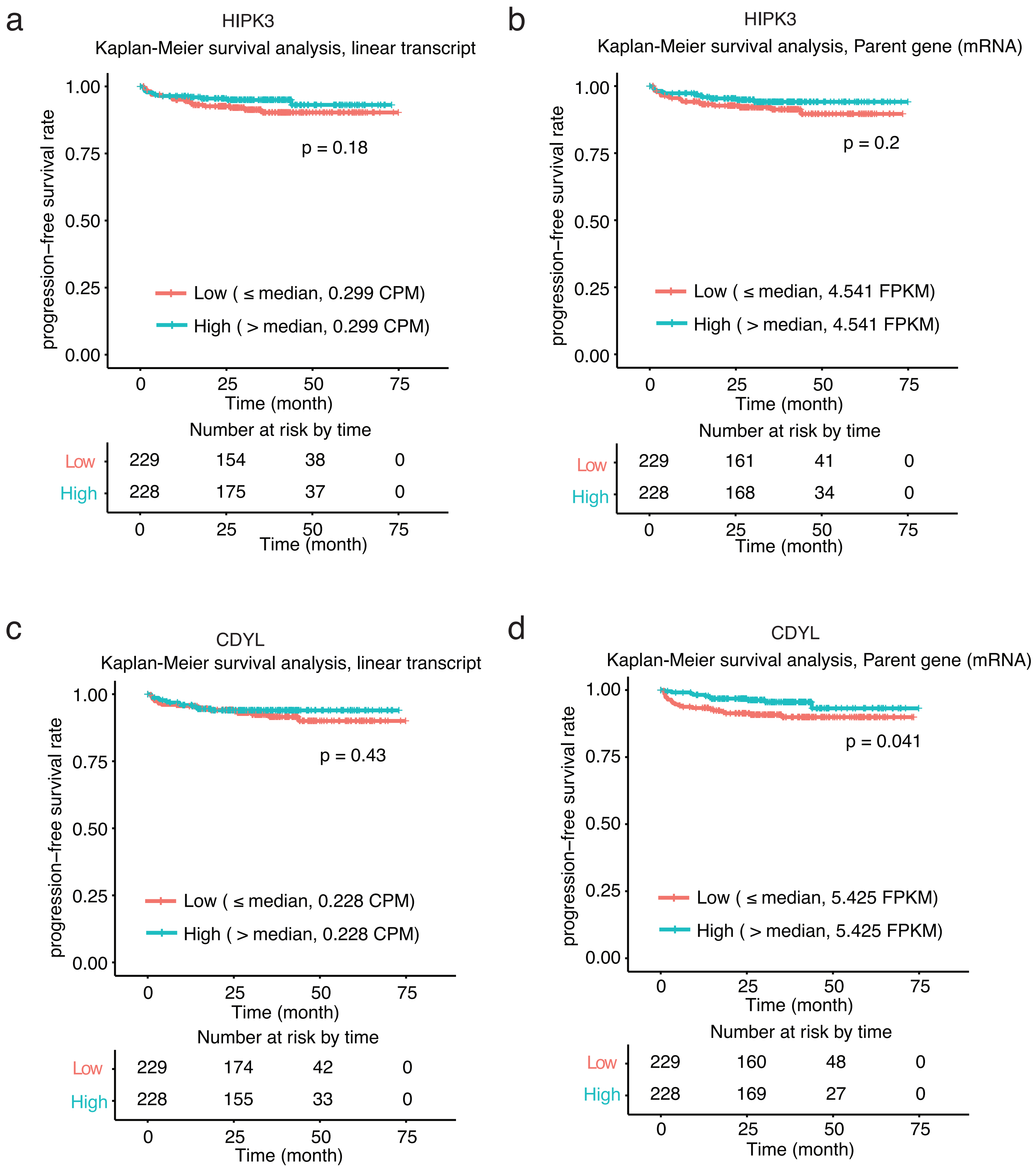

Supplementary Figure 8. Kaplan Meier plots of the linear counterpart and parent gene of circHIPK3 and circCDYL. Kaplan-Meier progression plots of **a** the linear counterpart of circHIPK3, **b** the gene HIPK3, **c** the linear counterpart of circCDYL, and **d** the gene CDYL. Median expression used as cutoff. P-values obtained by log-rank test.

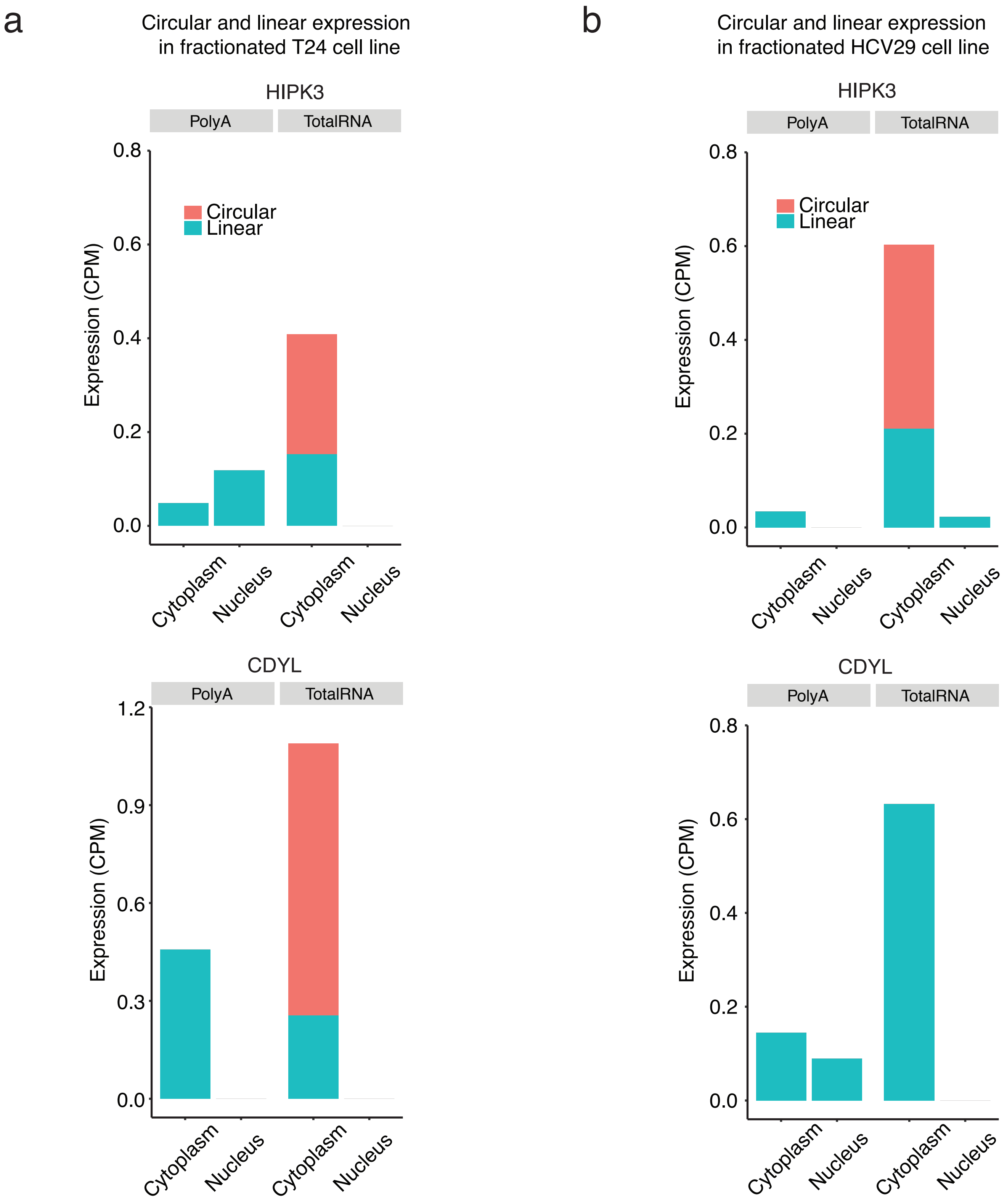

Supplementary Figure 9. Fractionated cell line expression. Circular and linear expression of HIPK3 and CDYL in the cytoplasm and nucleus of **a** T24 and **b** HCV29. Expression levels are measures based on either poly(A) selected RNAseq (PolyA; only mRNAs) or totalRNA (both mRNAs and circRNAs).
