# Supplementary material for: Circular RNA expression is abundant and correlated to aggressiveness in early-stage bladder cancer
Source: NPJ Genom Med. 2017 Nov 28;2:36. doi: 10.1038/s41525-017-0038-z (PMC5705701; doi:10.1038/s41525-017-0038-z)
Supplement: Supplementary file 6 — Supplementary Table 4 [file 41525_2017_38_MOESM6_ESM.pdf]

Feature	No. of circRNAs
Core splice site conservation (PhyloP score in 3rd or 4th quartile)	7/13
Inverted homologous Alu repeats in a window of 20,000 bp	10/13
Spliced from known cancer gene	0/13
Circular-to-linear ratio > 1	4/13
More observed SCEs than expected according to exon length	6/13
More observed miRNAs than expected according to exon length	4/13
CircRNA expression correlate with ADAR expression	1/13
CircRNA expression correlate with Quaking expression	12/13
CircRNA found in bladder cell lines	13/13
CircRNA expression correlate with EORTC score	5/13
CircRNA differentially expressed between risk class 1 and 2 (Wilcoxon Rank Sum Test)	9/13
Circular-to-linear ratio differentially expressed between risk class 1 and 2 (Wilcoxon Rank Sum Test)	8/13
Tissue expression (Expressed in more than 50% of 113 tissue samples)	10/13
